# Regulation of Nucleotide Excision Repair by UV-DDB: Prioritization of Damage Recognition to Internucleosomal DNA

**DOI:** 10.1371/journal.pbio.1001183

**Published:** 2011-10-25

**Authors:** Jia Fei, Nina Kaczmarek, Andreas Luch, Andreas Glas, Thomas Carell, Hanspeter Naegeli

**Affiliations:** 1 Institute of Pharmacology and Toxicology, University of Zürich-Vetsuisse, Zürich, Switzerland; 2 German Federal Institute for Risk Assessment (BfR), Department of Product Safety & Center for Alternatives to Animal Testing, Berlin, Germany; 3 Department of Chemistry and Biochemistry, Ludwig-Maximilian-University Munich, Munich, Germany; Erasmus University Rotterdam, Netherlands

## Abstract

How tightly packed chromatin is thoroughly inspected for DNA damage is one of the fundamental unanswered questions in biology. In particular, the effective excision of carcinogenic lesions caused by the ultraviolet (UV) radiation of sunlight depends on UV-damaged DNA-binding protein (UV-DDB), but the mechanism by which this DDB1-DDB2 heterodimer stimulates DNA repair remained enigmatic. We hypothesized that a distinctive function of this unique sensor is to coordinate damage recognition in the nucleosome repeat landscape of chromatin. Therefore, the nucleosomes of human cells have been dissected by micrococcal nuclease, thus revealing, to our knowledge for the first time, that UV-DDB associates preferentially with lesions in hypersensitive, hence, highly accessible internucleosomal sites joining the core particles. Surprisingly, the accompanying CUL4A ubiquitin ligase activity is necessary to retain the xeroderma pigmentosum group C (XPC) partner at such internucleosomal repair hotspots that undergo very fast excision kinetics. This CUL4A complex thereby counteracts an unexpected affinity of XPC for core particles that are less permissive than hypersensitive sites to downstream repair subunits. That UV-DDB also adopts a ubiquitin-independent function is evidenced by domain mapping and in situ protein dynamics studies, revealing direct but transient interactions that promote a thermodynamically unfavorable β-hairpin insertion of XPC into substrate DNA. We conclude that the evolutionary advent of UV-DDB correlates with the need for a spatiotemporal organizer of XPC positioning in higher eukaryotic chromatin.

## Abstract 2

This study reveals the molecular mechanism by which the nucleotide excision repair protein DDB2 prioritises excision of UV-induced DNA lesions in the nucleosome landscape.

## Author Summary

Like all molecules in living organisms, DNA undergoes spontaneous decay and is constantly under attack by endogenous and environmental agents. Unlike other molecules, however, DNA—the blueprint of heredity—cannot be re-created de novo; it can only be copied. The original blueprint must therefore remain pristine. All kinds of DNA damage pose a health hazard. DNA lesions induced by the ultraviolet (UV) component of sunlight, for example, can lead to skin aging and skin cancer. A repair process known as nucleotide excision repair (NER) is dedicated to correcting this UV damage. Although the enzymatic steps of this repair process are known in detail, we still do not understand how it copes with the native situation in the cell, where the DNA is tightly wrapped around protein spools called nucleosomes. Our study has revealed the molecular mechanism by which an enigmatic component of NER called UV-DDB stimulates excision of UV-induced lesions in the landscape of nucleosome-packaged DNA in human skin cells. In particular, we describe how this accessory protein prioritizes, in space and time, which UV lesions in packaged DNA to target for repair by NER complexes, thus optimizing the repair process.

## Introduction

Ultraviolet (UV) light generates mutagenic DNA lesions in the skin, primarily 6-4 pyrimidine-pyrimidone photoproducts (6-4PPs) and cyclobutane pyrimidine dimers (CPDs) [1] whose cytotoxic, inflammatory, and carcinogenic effects are mitigated by nucleotide excision repair (NER). Defects in this DNA repair system cause xeroderma pigmentosum (XP), a hereditary syndrome characterized by UV hypersensitivity and skin cancer [2],[3]. Although all principal biochemical steps are understood in detail [4]–[6], it is not yet known how NER is coordinated in the chromatin context, where the substrate is packed with histone proteins to generate arrays of nucleosome core particles joined by internucleosomal linkers [1],[7]. In the present study, we asked the question of how nucleosome arrays are inspected for DNA damage.

The UV-damaged DNA-binding (UV-DDB) and XPC-RAD23B complexes are the initial sensors of UV lesions in the global-genome repair branch of NER activity. XPC is essential for the recruitment of downstream NER factors including TFIIH, which comprises the XPB and XPD subunits, followed by XPA, replication protein A and the incision enzymes XPF-ERCC1 and XPG [8]. UV-DDB is a heterodimer: DDB1 associates with the CUL4A ubiquitin ligase [9]–[12], whereas DDB2 binds avidly to UV-irradiated DNA [13]–[18]. The absence of functional DDB2 in XP-E cells [19],[20] results in significantly delayed excision of 6-4PPs and overall reduced repair of CPDs [21],[22]. A widely accepted although unproven model is that UV-DDB recognizes these lesions and delivers the substrate to XPC, which is the actual NER initiator [22]–[26]. However, this putative handover remained elusive because it is not possible, for example in electrophoretic mobility shift assays, to detect stable intermediates where UV-DDB and XPC bind to the same damage simultaneously [23],[24],[27]. A general assumption was, therefore, that XPC is recruited only after the displacement of UV-DDB by CUL4A-mediated ubiquitylation and proteolysis [28]–[30]. The concomitant CUL4A-dependent ubiquitylation of XPC and histones is thought to potentiate the DNA-binding affinity of this repair initiator [25] and facilitate its access to chromatin [31],[32], but such models have been challenged by a more recent report where conditionally *CUL4A*-deleted mice show enhanced NER activity and resistance to UV-induced skin carcinogenesis [33]. Also, the known properties of UV-DDB have been difficult to reconcile with the manifestations of a *DDB2* mutation in XP-E patients because UV-DDB binds with highest affinity to 6-4PPs [34],[35], although it is required mainly for an effective CPD removal [21],[22]. However, reconstitution assays showed that UV-DDB is not at all needed for CPD excision from naked DNA [36], thus pointing to an as yet unidentified function in chromatin. Finally, it was difficult to understand why, after UV irradiation, DDB2 is degraded before the DNA lesions are fully repaired [29].

The aim of this study was to elucidate the so far enigmatic link between UV-DDB, XPC, and CUL4A by analyzing their crosstalk in the chromatin of living cells. We found a completely novel ubiquitin-dependent regulatory principle whereby UV-DDB inspects the nucleosome arrays to probe damaged chromatin for accessibility. Unexpectedly, the associated CUL4A ubiquitin ligase is required to retain the XPC partner at internucleosomal sites that are more permissive than the corresponding core particles to the assembly of downstream NER complexes. As a back-up function that is independent of chromatin localization and ubiquitin, the DDB2 subunit of UV-DDB associates transiently with the DNA-binding domain of XPC to fine-tune its engagement with CPD lesions.

## Results

### Hotspots of UV-DDB on Internucleosomal DNA

UV-DDB translocates to chromatin after UV irradiation [37]–[40], but this accessory sensor binds with highest affinity to 6-4PPs [35],[41] and earlier studies demonstrated that, in chromatin, 6-4PP lesions arise mainly in internucleosomal linker DNA between core particles [1],[42]. Prompted by these previous findings, we used a standard chromatin digestion assay to test the hypothesis that, in irradiated cells, UV-DDB accumulates preferentially at internucleosomal linker positions of nucleosome arrays. In particular, the localization of DDB2 (the DNA-binding subunit of UV-DDB) has been analyzed using the flow diagram of Figure S1A. First, free UV-DDB not bound to chromatin was removed by salt (0.3 M NaCl) extraction. Second, the resulting chromatin was dissected by a treatment with micrococcal nuclease (MNase). By cleaving internucleosomal linker regions (Figure S1B), this enzyme generates a solubilized supernatant representing digested internucleosomal sites (∼35% of cellular DNA), with traces of soluble core particles (∼5% of cellular DNA), and an insoluble fraction containing the vast majority of nuclease-resistant core particles (covering ∼60% of cellular DNA). This digestion pattern remained unchanged upon UV exposure as well as after siRNA-mediated DDB2 or XPC depletion and, in all cases, >80% of 6-4PPs appeared in MNase-sensitive internucleosomal regions whereas CPDs were evenly distributed across linker and core particle DNA (Figure S1C and S1D).

**Figure 1:**
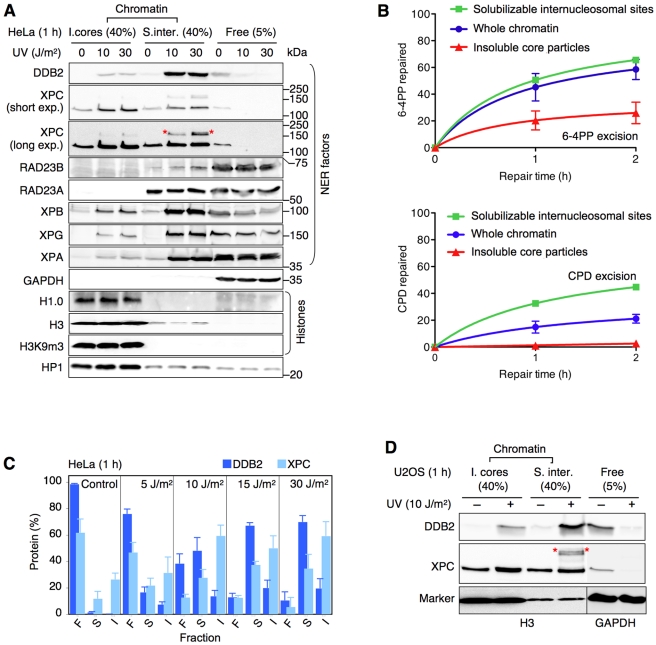
Preferential binding of UV-DDB to internucleosomal DNA. (A) The distribution of NER factors was analyzed in the chromatin of HeLa cells by MNase digestion (4 U/µl) and immunoblotting. GAPDH, glyceraldehyde-3-phosphate dehydrogenase; H3K9m3, trimethylated histone H3; HP1, heterochromatin protein 1. The asterisks in the long exposure denote ubiquitylated XPC. “I. cores,” insoluble core particles. “S. inter.,” solubilized internucleosomal sites. “Free,” proteins not bound to chromatin. The numbers in parentheses indicate the percentages of each fraction loaded onto the gel. (B) Initial excision of UV lesions from distinct nucleosomal sites measured by MNase treatment (4 U/µl) and enzyme immunoassay (average of three independent experiments). The repair of 6-4PPs and CPDs in digested internucleosomal DNA (squares) was calculated by subtracting the photolesions in core particles (triangles) from those found in whole genomic DNA (circles). The UV dose was 10 J/m^2^. (C) Quantification of the UV-dependent translocation of UV-DDB and XPC from the pool of free (F) proteins to solubilizable internucleosomal sites (S) and insoluble core particles (I). Relative amounts of DDB2 and XPC (mean values of 3–5 experiments) were calculated (see Text S1) from Western blot quantifications and corrections for differences in the loading volume as indicated in panel A. (D) UV-dependent relocation of DDB2 and XPC in p53-proficient U2OS cells determined by MNase digestion (4 U/µl).

As shown in Figure 1A, treatment of the chromatin of UV-irradiated cells with a saturating MNase concentration (4 U/µl), which digests all linker DNA, released ∼70% of total DDB2 into the solubilized internucleosomal fraction (“S. inter.”) and only ∼20% of the cellular DDB2 pool remained associated with insoluble core particles (“I. cores”). In dose dependence experiments, even low MNase concentrations, which resulted in mild DNA digestions, liberated the same amount of DDB2 from chromatin (Figure S1E), thus confirming that UV-DDB binds predominantly to nuclease-hypersensitive and, hence, highly accessible internucleosomal DNA. These UV-DDB- and 6-4PP-enriched sites coincide with NER hotspots, as they were more permissive than insoluble core particles to the UV-dependent recruitment of downstream NER subunits like XPB (a TFIIH subunit), XPA, and XPG (Figure 1A). The accumulation of NER factors at these solubilizable internucleosomal sites led to faster kinetics of 6-4PP and CPD excision, measured by an immunoassay procedure, in comparison to the slow removal of these lesions from core particles (Figure 1B).

Unlike UV-DDB, XPC displayed a constitutive binding to both MNase fractions of chromatin even in the absence of UV lesions. However, in response to DNA damage, XPC moved by a large extent to the MNase-resistant and slowly repaired core particles (Figure 1A and 1C). Such a preferential XPC binding to core particles, accompanied by a UV-DDB translocation mainly to solubilizable internucleosomal sites, was also observed in p53-proficient U2OS fibroblasts (Figure 1D). The much higher amount of histone H3 as well as a co-localization of trimethylated H3 (H3K9m3), histone variant H1.0, and heterochromatin protein 1, which correlate with chromatin condensation [43],[44], support the conclusion that this insoluble fraction contains the bulk of nucleosome core particles. Importantly, the sequestration of XPC on these core particles reflects a specific binding to histone-assembled DNA, rather than the formation of insoluble protein aggregates, as the removal of core histones with 2.5 M NaCl [45] resulted in a nearly complete XPC release (Figure S1F).

### Distinct Features of XPC in Different Nucleosome Microenvironments

**Figure 2:**
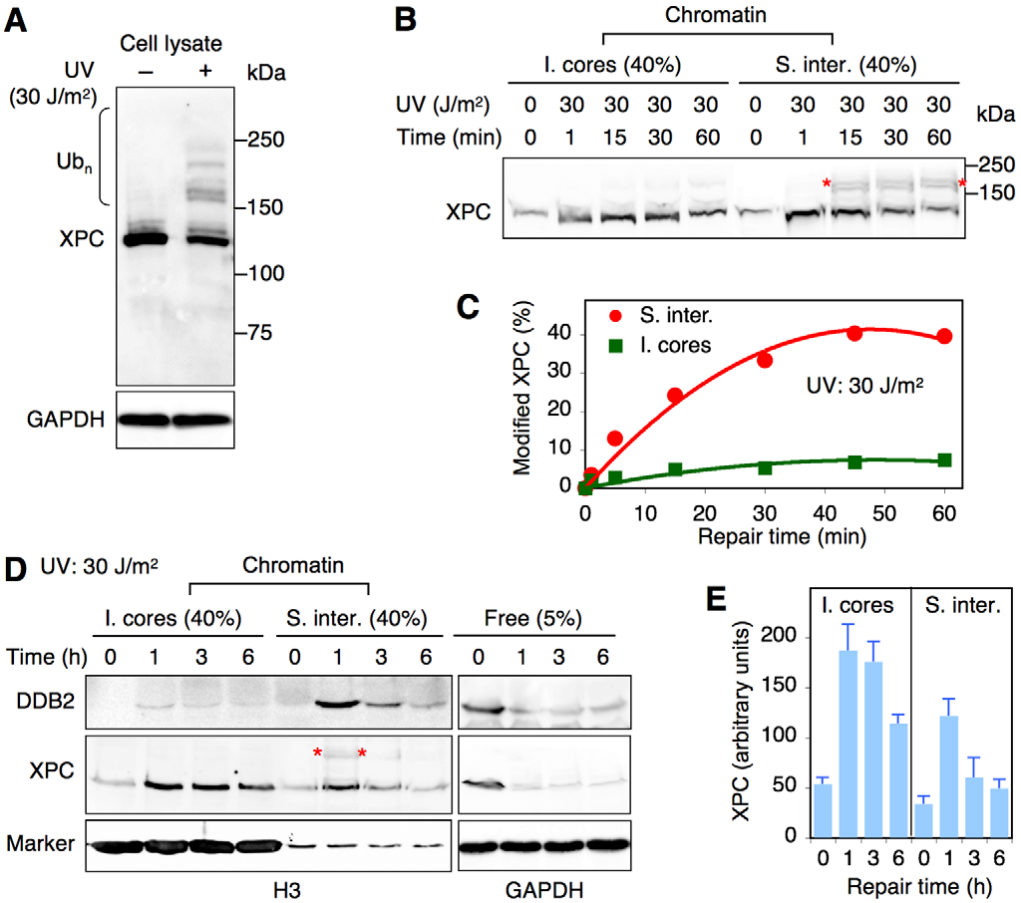
Differential XPC ubiquitylation in chromatin. (A) UV-dependent ubiquitylation of XPC protein visualized by an immunoblot of HeLa whole-cell lysates 15 min after UV irradiation (30 J/m^2^). Ub_n_, ubiquitylated forms of XPC. (B) The chromatin of HeLa cells was dissected by MNase digestion (4 U/µl) at different times after UV exposure to compare the ubiquitylation of XPC bound to the core particle fraction (“I. cores”) or internucleosomal DNA (“S. inter.”). (C) Quantitative comparison of ubiquitylated XPC relative to total XPC associated with either solubilizable (4 U/µl) internucleosomal sites (“S. inter.”) or MNase-resistant core particles (“I. cores”) at the indicated times after UV irradiation (mean values of two independent determinations). (D) Representative blot illustrating the DDB2 degradation and long-term binding of XPC to the insoluble core particle fraction (“I. cores”) after UV exposure. (E) Quantification of the time-dependent XPC distribution in the chromatin of UV-exposed HeLa cells. XPC amounts in the core particle fraction (“I. cores”) and at internucleosomal sites (“S. inter.”) were calculated from Western blots followed by corrections for the different loading as indicated in panel D (mean values of three independent determinations).

Several parameters distinguish the just described MNase-solubilizable internucleosomal sites and MNase-resistant core particles. First, immunoblots against XPC revealed multiple higher molecular weight forms (>150 kDa), known to occur by polyubiquitylation [25],[46], that begin appearing within ∼5 min after UV irradiation (Figure 2A). It is important to note that, by increasing the polyacrylamide concentration, this typical ladder-like appearance of ubiquitylated XPC molecules was compressed to a more discrete signal in most immunoblots of this report. We consistently found that the proportion of ubiquitylated XPC, relative to unmodified protein, is markedly increased on internucleosomal DNA compared to the slowly repaired core particles (Figure 2B). Up to 40% of XPC bound to solubilizable internucleosomal sites but <10% in insoluble core particles are modified (Figure 2C). The substantial, although not complete, separation of ubiquitylated and non-ubiquitylated species achieved by MNase digestion suggested that this modifier plays a role in regulating the XPC partitioning within nucleosome repeats of chromatin (see siRNA-mediated depletion assays below).

A second difference was disclosed when the same samples were probed with antibodies against RAD23B. As observed in cell extracts, where XPC is mainly complexed with RAD23B [47], XPC carried this interaction partner to internucleosomal sites. However, the fraction of XPC that associated with the slowly repaired core particles is not accompanied by RAD23B (Figure 1A). For comparison, RAD23A (the second RAD23 homolog) is found only at internucleosomal sites independently of a UV stimulus. The third difference concerns the time course of XPC accumulation. In fact, XPC relocated to internucleosomal DNA immediately after UV irradiation (Figure 2B, 1-min time point) and, in this rapidly repaired microenvironment, returned to background levels corresponding to the constitutive XPC binding to chromatin within ∼3 h (Figure 2D). Instead, the UV-dependent XPC recruitment to insoluble core particles persisted further, thus reflecting a long-term DNA repair response. After an incubation of 6 h following irradiation, when DDB2 is reduced to ∼20% of its pre-irradiation level due to proteolytic degradation (Figure S2A) [29], the majority of chromatin-bound XPC was sequestered on these slowly repaired core particles (Figure 2D and 2E). Thus, time course experiments suggested that DDB2 is important to retain high levels of XPC on internucleosomal DNA (see siRNA-mediated depletion assays below).

### Ubiquitin-Dependent XPC Partitioning in Nucleosome Arrays

**Figure 3:**
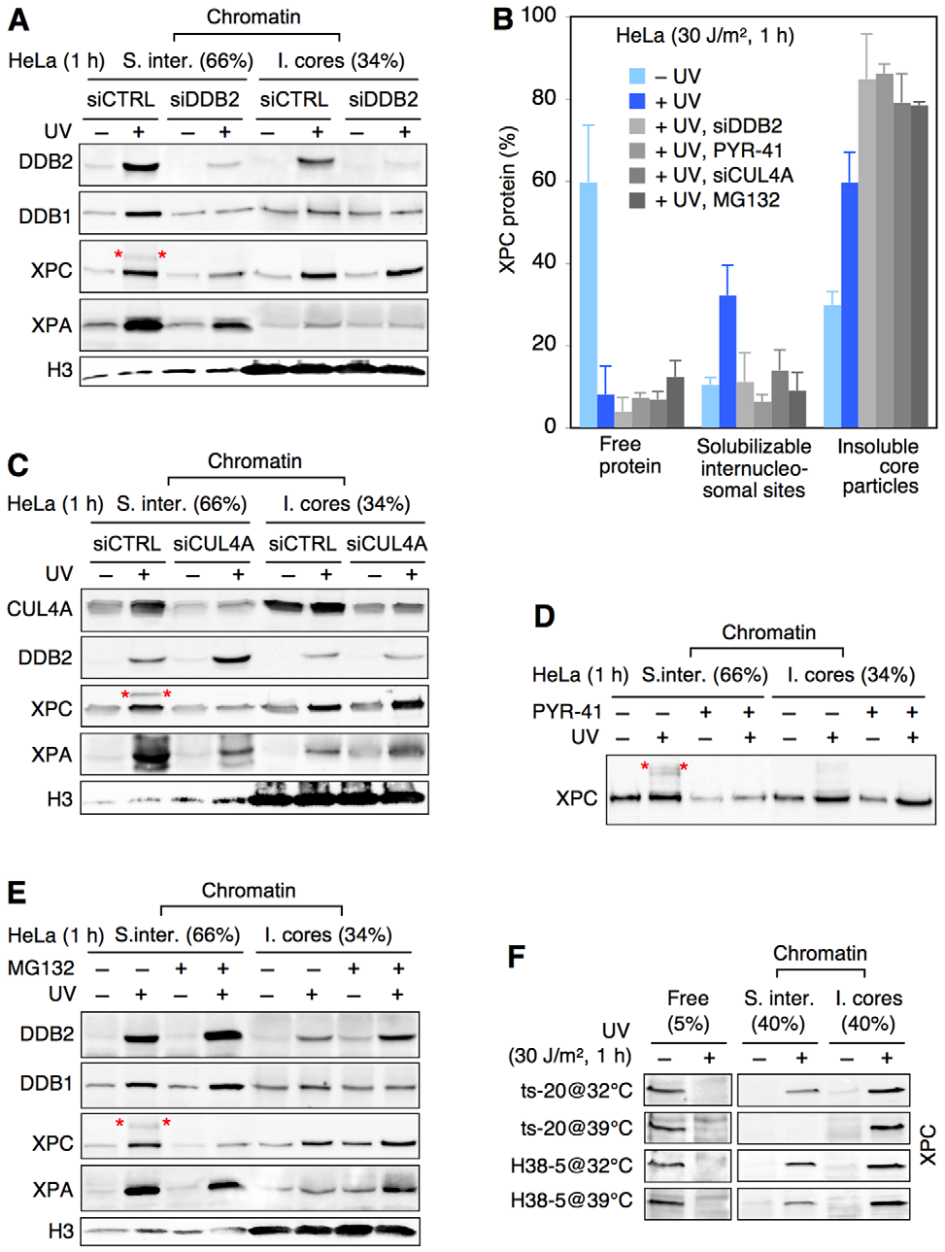
Ubiquitin-dependent XPC retention on internucleosomal DNA. (A) Comparison between internucleosomal sites (“S. inter.”) and core particles (“I. cores”) illustrating that a DDB2 depletion alters the chromatin distribution of DDB1, XPC, and XPA. DDB2 was down regulated by transfection with siRNA and the chromatin was dissected by MNase digestion (4 U/µl) 1 h after UV irradiation (30 J/m^2^); siCTRL, control RNA. (B) Quantitative assessments demonstrating the abnormal XPC distribution (reduced retention at solubilizable internucleosomal sites and increased binding to insoluble core particles) after inhibition of the ubiquitylation pathway by different treatments. The XPC translocation to the indicated nucleosome fractions (mean values of 3–5 experiments) was determined from Western blots with corrections for the differences in loading as indicated in panel A (see Text S1). (C) Representative blot illustrating the altered XPC and XPA distribution following CUL4A depletion (UV: 30 J/m^2^). (D) Representative blot showing that a treatment with the E1 inhibitor PYR-41 reduces the XPC retention at internucleosomal sites without diminishing its UV-dependent accumulation in the core particle fraction. The UV dose was 30 J/m^2^. (E) Representative blot illustrating that MG132 reduces the UV-dependent XPC retention and the subsequent recruitment of XPA to internucleosomal sites (UV: 30 J/m^2^). (F) E1 inactivation in ts-20 cells (at 39°C) suppresses the UV-dependent XPC retention at internucleosomal sites (“S. inter.”). This response is not observed at 32°C or in corrected H38-5 cells. The available antibodies are unable to detect ubiquitylated mouse XPC.

As expected, the preferential appearance of DDB2 (the DNA-binding subunit of UV-DDB) on internucleosomal DNA was accompanied by an equivalent accumulation of DDB1 (its regulatory adaptor) in response to UV light. A DDB2 depletion by transfection with specific siRNA (Figure S2B) prevented this UV-induced DDB1 translocation to chromatin and, accordingly, suppressed the ubiquitylation of XPC (Figure S2C). As a consequence of this diminished ubiquitylation, the relocation of XPC to internucleosomal sites, but not to insoluble core particles, was reduced (Figure 3A). This and follow-up findings involving the role of protein ubiquitylation are confirmed by a quantitative assessment of immunoblots over 3–5 independent experiments (Figure 3B). In siRNA-mediated depletion experiments, DDB2 was down regulated incompletely to ∼10% of control cells (Figure S2B). However, a stronger aversion of XPC for internucleosomal DNA was observed in XP-E cells displaying no residual UV-DDB activity (Figure S2D). Finally, Figure S2E shows that the normal abundance of XPC at solubilizable internucleosomal sites was restored upon complementation of DDB2-depleted cells with DDB2 fused to green-fluorescent protein (DDB2-GFP).

**Figure 4:**
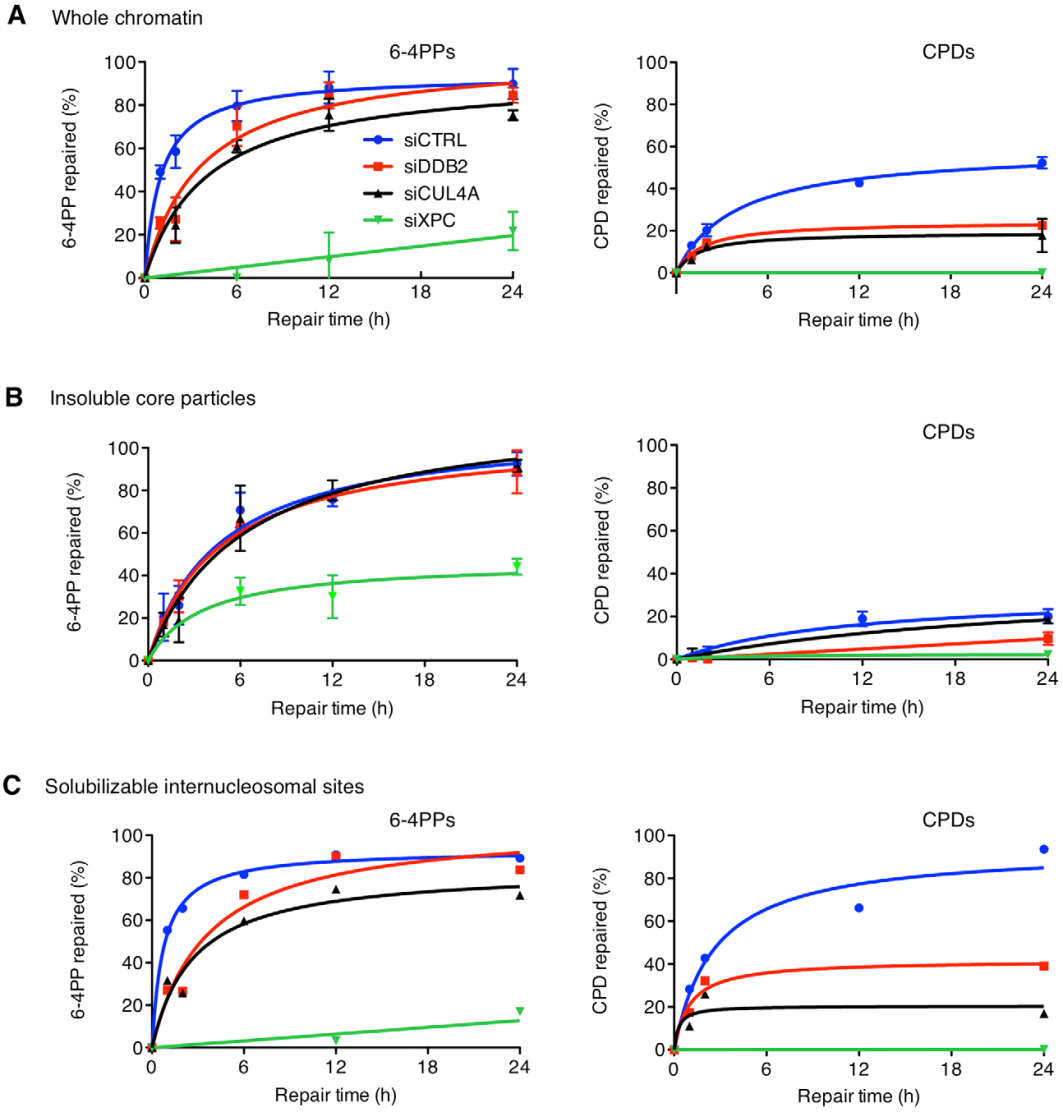
CUL4A-dependent excision of UV lesions from internucleosomal DNA. The excision of 6-4PPs (*left*) and CPDs (*right*) was determined in whole chromatin (A) and MNase-insoluble (4 U/µl) core particles (B) of HeLa cells transfected with the indicated siRNA reagents and exposed to UV light (10 J/m^2^). Relative amounts of each photolesion were determined at the indicated times by enzyme immunoassay (mean values of 3–5 independent determinations). Subsequently, the excision of UV lesions from MNase-solubilizable internucleosomal DNA (C) was calculated by subtracting the photolesions in the insoluble core particle fraction from those found in the whole chromatin. The initially delayed 6-4PP excision in a DDB2-depleted background (siDDB2) fits with the reported repair kinetics of XP-E cells [21],[22].

CUL4A is primarily responsible for XPC ubiquitylation, while CUL4B (the other CUL4 family member) plays essentially no role in this process [33]. Therefore, to provide a direct proof for the function of ubiquitin modifiers in XPC positioning, four different strategies were used to dissociate UV-DDB from the CUL4A machinery. As expected, a siRNA-mediated CUL4A depletion (Figure S2B) suppressed XPC ubiquitylation (Figure S2C) and increased the DDB2 level in chromatin by preventing its UV-dependent proteolytic degradation (Figure 3C). Consistent with the just described effects of a DDB2 down regulation, the missing CUL4A activity reduced the presence of XPC at internucleosomal sites, but not in the insoluble core particle fraction, thus limiting the overall recruitment of downstream subunits like XPA to UV-irradiated chromatin (Figure 3C). Accompanying UV lesion excision assays demonstrated that this CUL4A depletion mimics the effect of a DDB2 deficiency by delaying substantially the removal of 6-4PPs and inhibiting the overall CPD repair (Figure 4A). However, in the corresponding core particles, this CUL4A depletion had no effect on 6-4PP excision and caused only a marginal, if any, further reduction of the slow rate of CPD removal (Figure 4B). As illustrated in Figure 4C, these functional assays therefore reveal that the CUL4A ubiquitin ligase is needed primarily for an effective DNA repair of internucleosomal sites, where its depletion slows down substantially the fast excision of 6-4PPs and strongly inhibits the processing of CPDs.

Next, we confirmed these effects of a DDB2 or CUL4A down regulation using small-molecule inhibitors. The E1 inhibitor PYR-41 suppressed XPC ubiquitylation following UV exposure (Figure S3A) and, as a consequence, inhibitor-treated cells were unable to retain XPC at internucleosomal sites upon UV irradiation. In contrast, the UV-dependent XPC accumulation in the core particle fraction was unchanged (Figure 3B and 3D). The proteasome inhibitor MG132 raised the DDB2 level in chromatin by inhibiting its UV-dependent proteolytic degradation. In addition, by depletion of the free ubiquitin pool, MG132 impedes the ubiquitylation of nuclear substrates [48] including XPC (Figure S3B). As a consequence of this MG132-inhibited ubiquitylation, XPC failed to persist at internucleosomal sites but was still able to bind to core particles (Figure 3B and 3E). Time course experiments with MG132 confirmed the finding of Figure 2B (1-min time point) demonstrating that the initial UV-dependent shuttling of XPC to internucleosomal sites is completely independent of ubiquitin. However, the subsequent ubiquitylation is required to retain XPC on these internucleosomal DNA locations (Figure S3C). As DDB2 and p53 regulate the synthesis of one another [21],[49], the MG132 inhibitor has also been used to confirm the key role of ubiquitylation in retaining XPC at internucleosomal sites in p53-proficient U2OS cells (Figure S3D).

Finally, this ubiquitin function was further established using mouse cells that harbor a temperature-sensitive ubiquitin-activating E1 enzyme [25],[46]. Due to their ubiquitylation defect when incubated at 39°C, these ts20 cells are unable to retain XPC at internucleosomal sites and, hence, respond to UV light with a nearly complete XPC translocation to the insoluble core particle fraction (Figure 3F). Instead, in control H38-5 cells corrected with wild-type E1, XPC was effectively retained at solubilizable internucleosomal sites at both 32°C and 39°C.

### Ubiquitin-Independent UV-DDB Function

**Figure 5:**
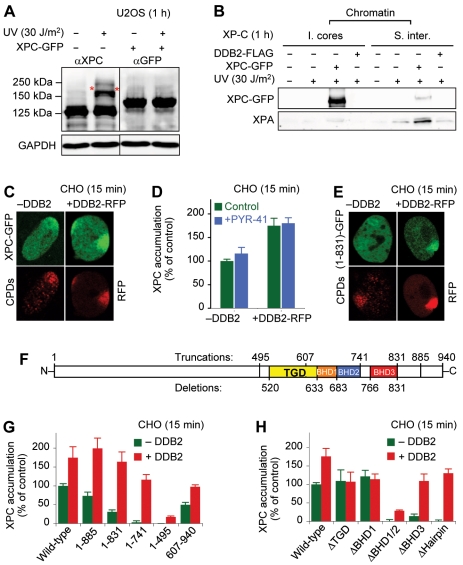
Ubiquitin-independent XPC recruitment by UV-DDB. (A) Minimal ubiquitylation of XPC-GFP, compared to endogenous XPC, demonstrated by Western blotting. (B) UV-dependent binding of XPC-GFP primarily to MNase-insoluble (4 U/µl) core particles (“I. cores”) of XP-C fibroblasts. The subsequent XPC-dependent recruitment of XPA occurs mainly to solubilizable internucleosomal DNA (“S. inter.”). (C) XPC-GFP relocation to UV-irradiated areas of DDB2-deficient CHO cells. UV lesion spots were visualized with CPD antibodies or by monitoring DDB2-RFP intensity. (D) XPC-GFP relocation to UV lesions stimulated by co-expression of DDB2-RFP. GFP signals at UV lesion spots (*N* = 30) were quantified, normalized against the nuclear background, and expressed as a percentage of controls (XPC alone). (E) DDB2-stimulated relocation of XPC_1–831_-GFP to UV-irradiated areas. (F) Domain structure of human XPC (see Figure S4B for truncations and deletions). (G) Recruitment of XPC-GFP truncates. Fluorescence spots co-localizing with UV lesions (*N* = 30) were normalized and expressed as a percentage of control values (full-length XPC alone). (H) Recruitment of XPC-GFP deletions to UV lesions (*N* = 30).

To search for direct UV-DDB actions, not mediated by ubiquitin, we exploited an XPC-GFP fusion that, unlike endogenous XPC, was poorly ubiquitylated (Figure 5A). Following 1 h after UV irradiation, a minor but detectable proportion of this construct remained at internucleosomal sites (Figure 5B) where it led to recruitment of downstream NER effectors like XPA, thus explaining its ability to correct the UV hypersensitivity of XP-C cells [47]. However, consistent with its poor susceptibility to ubiquitylation, most of these XPC-GFP constructs associated with the insoluble core particle fraction (Figure 5B) as noted before (Figure 3) for endogenous XPC in the background of a defective UV-DDB-CUL4A pathway.

To monitor DDB2-XPC interactions within chromatin rather than as free proteins in solution, this poorly ubiquitylated XPC-GFP fusion was expressed in Chinese hamster ovary (CHO) cells that lack endogenous DDB2 [50]. After local damage induction by irradiation through polycarbonate filters [39], during which only parts of each nucleus are exposed to UV light, we measured the increase of green fluorescence intensity in irradiated areas over the surrounding nuclear background. Figure 5C illustrates that the UV-dependent XPC-GFP accumulation was enhanced by co-expression of DDB2, which was tagged with red-fluorescent protein (DDB2-RFP). Time course experiments showed that the accumulation of XPC reaches a maximum around 15 min after irradiation (Figure S4A). Importantly, the stimulation of lesion recognition by DDB2 was insensitive to the E1 inhibitor PYR-41 (Figure 5D), thus confirming the notion that, by this approach, we measured a ubiquitin-independent UV-DDB function. Also, this stimulation of lesion recognition was maintained with an XPC truncate (XPC_1–831_) that, on its own, binds weakly to damaged sites (Figure 5E), indicating that a DNA-independent association between UV-DDB and XPC is involved in the substrate handover between these two factors.

Next, the filter irradiation assay was used to map UV-DDB-XPC interactions in chromatin using the constructs outlined in Figures 5F and S4B. Compared to full-length XPC, the truncate XPC_1–741_, like XPC_1–831_, showed a defective relocation to damaged sites but was still attracted to UV lesions when co-expressed with DDB2-RFP. Instead, the N-terminal fragment XPC_1–495_ was recruited to UV damage sites less efficiently than the full-length control or the much shorter C-terminal fragment XPC_607–940_ (Figure 5G). Collectively, this in situ mapping suggested that XPC residues 496–741, comprising a transglutaminase homology domain (TGD) and parts of the β-hairpin domains (BHDs), associate with DDB2. By eliminating the respective sequences, we tested the individual contribution of each of these motifs to DDB2-XPC interactions. TGD-deleted (ΔTGD) and BHD1-deleted (ΔBHD1) constructs display the same damage recognition capacity as the full-length control, but their accumulation in UV foci was not stimulated by co-expression of DDB2 (Figure 5H). In contrast, the BHD3 sequence is dispensable for DDB2-XPC interactions because the ΔBHD3 deletion construct was still efficiently recruited to UV lesions by DDB2 (Figure 5H).

### DDB2-XPC Contacts Stimulated by DNA Damage

We characterized the ubiquitin-independent UV-DDB-XPC associations by transfecting HEK293T cells with DDB2-FLAG and XPC-GFP fusions, followed by co-immunoprecipitation using anti-FLAG antibodies (Figure S4C). In the presence of full-length DDB2_1–427_-FLAG, the isolated complexes comprised both endogenous DDB1 and XPC-GFP, demonstrating that there was sufficient free cellular DDB1 to probe its role in these interactions (Figure S4D). Additional co-immunoprecipitations showed that an N-terminal DDB2 truncate (DDB2_79–427_-FLAG), which failed to associate with DDB1, still bound efficiently to XPC-GFP, demonstrating that DDB1 is not implicated in this binary DDB2-XPC crosstalk. The co-immunoprecipitations with fusion fragments XPC_520–633_-GFP and XPC_607–831_-GFP provided further support to the notion that DDB2 associates with both the TGD (Figure S4E) and BHD regions of XPC (Figure S4F).

**Figure 6:**
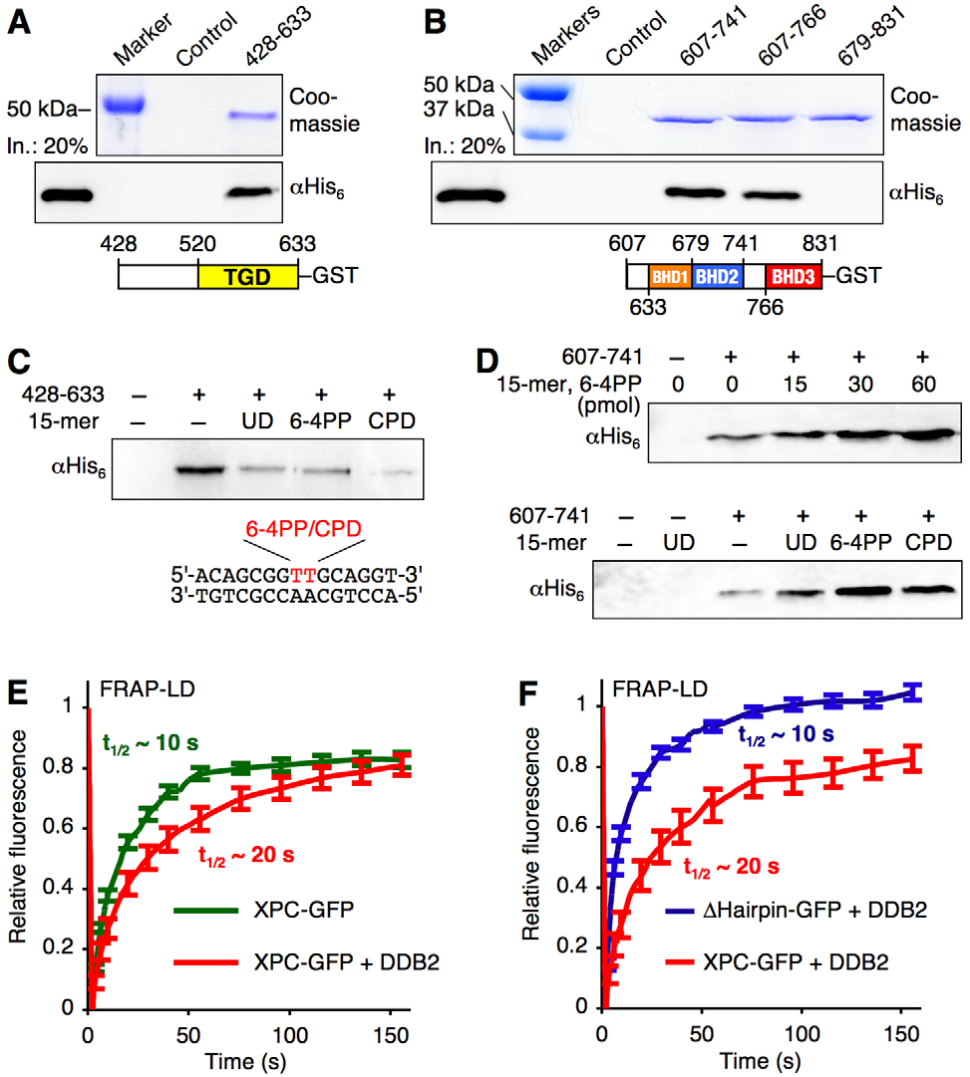
Dynamic DDB2-XPC interactions in chromatin. (A) DDB2 association with the TGD motif of XPC. Purified UV-DDB-His_6_ (120 pmol) was incubated with XPC_428–633_-GST (120 pmol) and probed by adding glutathione beads. XPC_428–633_-GST in the pull-down fraction was visualized by Coomassie staining whereas antibodies against His_6_ detected interacting DDB2. An input (“In.”) control displays 20% of total DDB2 in each incubation. (B) DDB2 association with XPC_607–741_-GST and XPC_607–766_-GST, both containing the BHD1/BHD2 motifs. (C) Interaction between DDB2 and XPC_428–633_-GST inhibited by a 15-mer DNA duplex (120 pmol). UD, undamaged. The 15-mer sequence is shown with the 6-4PP or CPD position in red. (D) Interactions between DDB2 and XPC_607–741_-GST are stimulated by a 15-mer DNA duplex (top panel: 15–60 pmol; bottom panel: 120 pmol). (E) Dissociation of XPC-GFP from UV lesions in CHO cells measured by FRAP-LD (*N* = 15; ± s.e.m.). Half-lifes were estimated from the fluorescence recovery curves. (F) DDB2 is unable to stabilize the ΔHairpin deletion at UV lesions in FRAP-LD assays (*N* = 15). A control fluorescence recovery in the absence of DDB2 is not possible because the ΔHairpin deletion alone fails to relocate to DNA damage.

In view of this preliminary domain mapping in HEK293T cells, polypeptides containing the TGD (XPC_428–633_), BHD1/2 (XPC_607–741_), or BHD2/3 (XPC_679–831_) sequences were tested as purified glutathione-*S*-transferase (GST) fusions, thus demonstrating that the TGD (Figure 6A) and BHD1/2 motifs (Figure 6B) make direct contacts with DDB2. In contrast, a polypeptide of similar length comprising the BHD2/3 sequence did not associate with DDB2, thus excluding this part of XPC as the interaction surface. We next found that DDB2-TGD associations are inhibited by the addition of either undamaged or damaged double-stranded DNA (Figure 6C). This latter finding provides a plausible explanation for the fact that it has never been possible to isolate and characterize a stable ternary complex with simultaneous binding of both UV-DDB and XPC to substrate DNA [23],[27].

In contrast to this interaction with the TGD motif, the association of DDB2 with the BHD1/2 fragment was stimulated by short DNA duplexes carrying a site-specific lesion. In line with the distinct affinity of UV-DDB for different types of UV damage, DNA duplexes with a 6-4PP promoted this interaction more efficiently than those carrying a CPD (Figure 6D). Taken together, these results indicate a dynamic process whereby the DDB2 subunit of UV-DDB first recruits XPC through a DNA-independent association with TGD and then positions XPC onto the lesion site by a DNA damage-stimulated interaction with BHD1.

### Dynamic DDB2-XPC Handover

The identification of an XPC domain, whose association with UV-DDB is stimulated by damaged DNA, demonstrated that the two factors are able to bind transiently to the same lesion. To understand how damaged DNA is transferred from UV-DDB to XPC during ongoing repair, we transfected CHO cells with XPC-GFP, alone or in combination with DDB2-RFP. Following the induction of local UV damage by irradiation through polycarbonate filters, the in situ stability of XPC-DNA interactions was tested by bleaching the green fluorescence signal at damaged sites, thus reducing its intensity to that of the surrounding nuclear background [51],[52]. The subsequent fluorescence recovery due to exchanges of bleached molecules with non-bleached counterparts was recorded over time, thus yielding distinct dissociation curves. In fact, this real-time analysis of nucleoprotein stability by fluorescence recovery after photobleaching on local damage (FRAP-LD) revealed that most XPC is only transiently immobilized at DNA lesions and that the expression of DDB2 doubles the half-life of these dynamic interactions between XPC and damaged DNA from ∼10 s to ∼20 s (Figure 6E). Conversely, the dissociation of DDB2, tested as a GFP fusion, from UV lesions was accelerated by XPC (Figure S5A).

Ultimately, damage recognition by XPC involves the insertion of a β-hairpin of BHD3 into the DNA double helix [53]. To test the role of this key rearrangement during the UV-DDB-XPC handover, we constructed an appropriate deletion by removing residues 789–815 from the human XPC sequence. The resulting β-hairpin-deleted mutant (ΔHairpin), although unable to detect DNA damage on its own, was very effectively recruited to UV lesions upon co-expression with DDB2 (see Figure 5H). Next, this ΔHairpin construct that relocates to damage in the presence of DDB2 has been subjected to FRAP-LD analyses to test again the in situ half-life of its interactions with substrate DNA. The resulting steep slope of fluorescence redistribution indicated, however, that UV-DDB fails to stabilize the ΔHairpin binding to damaged DNA (Figure 6F). Also, the dissociation of DDB2 from damaged DNA was not accelerated by this ΔHairpin deletion (Figure S5B). Thus, although UV-DDB attracts XPC to lesion sites, it only prolongs its residence time at damaged targets if XPC itself is able to insert the β-hairpin subdomain into the substrate double helix.

## Discussion

Since the identification of UV-DDB as an accessory DNA damage sensor, this heterodimer has been the subject of intense scrutiny, but its mechanism of action remained elusive. A consensus model is that UV-DDB helps to recruit the XPC partner to UV lesions [38],[39],[54]. However, experimental evidence for the suggested handover from UV-DDB to XPC is lacking because it has not been possible to isolate and characterize nucleoprotein intermediates where these two factors bind jointly to the same DNA substrate [23],[24],[27]. As to the associated CUL4A complex, it is generally thought that this ubiquitin ligase promotes the removal of UV-DDB from damaged sites [25],[29],[30], enhances the DNA-binding affinity of XPC [25] or opens chromatin to facilitate UV lesion recognition [31],[32]. After reexamining this long-standing issue in the nucleosome context of living cells, we now present an unexpected function that fully accommodates the role of UV-DDB and CUL4A in stimulating DNA excision repair. We found that UV-DDB inspects the chromatin to detect lesions preferentially, although not exclusively, in highly accessible internucleosomal sites distinguishable by their MNase hypersensitivity, and that the accompanying CUL4A-mediated ubiquitylation serves to retain the XPC partner at these particularly permissive DNA repair hotspots.

### A Novel Regulatory Role for the CUL4A Ligase

This newly identified UV-DDB and CUL4A function is critical for effective DNA repair because XPC, the initiator of NER activity, otherwise binds primarily to nucleosome core particles that represent a less permissive environment characterized by (i) poor recruitment of downstream NER subunits and (ii) slow excision of UV lesions (Figure 1). This property of XPC, i.e. its default-mode association with damaged core particles in the whole-chromatin context, challenges a long-held notion derived from biochemical reconstitution experiments [55],[56] that nucleosome repeats pose a barrier to recognition of UV lesions by XPC.

Interestingly, the characteristic XPC binding to damaged core particles is independent of UV-DDB- and CUL4A-mediated ubiquitylation (Figure 3). We even observed that, upon exposure to UV light, the initial XPC accumulation on internucleosomal DNA does not require the ubiquitylation reaction (Figures 2B and S3C). However, the following ubiquitin modification is essential to retain XPC at these highly accessible internucleosomal positions that allow for the fast excision of both 6-4PPs (half-life in internucleosomal DNA ∼1 h) and CPDs (half-life in internucleosomal DNA ∼2 h) (Figure 1B). It is important to point out that 6-4PPs are generated with ∼8-fold higher density in internucleosomal sites than in core particles [1],[42]. Thus, the fast CUL4A-dependent excision from internucleosomal DNA accounts for nearly all global repair of this lesion across the genome. As summarized in Figure 3B, the ubiquitin-dependent retention of XPC at internucleosomal sites is abolished by depletion of DDB2 or CUL4A, by inhibition of the E1 ubiquitin-activating enzyme (using a small-molecule inhibitor or a temperature-sensitive mutant), or by depletion of the ubiquitin pool (using a proteasome inhibitor).

That the chromatin location of XPC is determined by its own CUL4A-dependent modification can be inferred from an XPC-GFP fusion, which is poorly polyubiquitylated (although monoubiquitylation cannot be completely ruled out) and whose chromatin partitioning, characterized by a strong binding to damaged core particles, is similar to that observed with endogenous XPC after blocking the ubiquitylation pathway (Figure 5B). Despite such a negative effect exerted by the GFP tag on the CUL4A machinery, this construct complements the overt hypersensitivity of XP-C cells to killing by UV radiation [47] and, in our study, provides a helpful tool to demonstrate that it is the ubiquitylation of XPC itself that fine-tunes the nucleosome partitioning of this repair initiator. The resulting ubiquitin-dependent retention at internucleosomal sites may be a consequence of an increased affinity of polyubiquitylated XPC for naked DNA as reported by Sugasawa et al. (2005) [25]. Conversely, the lack of ubiquitin modifications may favor the release of RAD23B because we noted with two different antibodies that non-ubiquitylated XPC, which binds to core particles, is separated from RAD23B (Figure 1A). By mediating CUL4A activity, UV-DDB not only controls the spatial distribution of XPC but also the differential timing of its dissociation from chromatin. Indeed, the concomitant proteolysis of DDB2, induced by CUL4A, terminates the just described XPC retention at internucleosomal sites. With progressive DDB2 degradation after UV exposure, a growing proportion of chromatin-associated XPC evades ubiquitylation and, hence, disappears from internucleosomal DNA (Figure 2D and 2E).

### A Dynamic Platform for CPD Recognition

The results discussed so far explain the delayed excision of UV lesions from internucleosomal sites in a DDB2- or CUL4A-deficient background (Figure 4C). Yet they do not accommodate the very slow removal of CPDs from nucleosome core particles following a DDB2 depletion, particularly considering that a comparable CUL4A depletion does not significantly affect the excision of these lesions from the same core particle substrate (Figure 4B). In support of a CUL4A-independent action, we found that, in addition to associating with the DDB1-CUL4A machinery, the DDB2 subunit makes direct contacts with a region of XPC that overlaps partly with its DNA-binding surface. The evidence underlying this conclusion is that DDB2 stimulates the recruitment of XPC-GFP fusions to UV lesions and that this recruitment is not affected by inhibition of the ubiquitylation pathway. Direct interactions are made between DDB2 and the TGD and BHD1 regions, two neighboring DNA-binding motifs of XPC (Figure 6). An association with TGD occurs regardless of DNA, whereas the binding to BHD1 is stimulated by damaged substrates, indicating that DDB2 and XPC alternate their contacts to hand over the DNA lesion from one recognition factor to the next.

The relevance of these direct interactions is demonstrated by ΔTGD and ΔBHD1 deletions whose recruitment to DNA damage is not stimulated by DDB2 (Figure 5H). In situ analyses of the role of these domains by protein dynamics show that damage-specific DDB2-XPC interactions take place transiently, that they stabilize the association of XPC with UV lesions, and that this stabilization additionally depends on a β-hairpin subdomain located in BHD3 (Figure 6). Because DDB2 does not make physical contacts with this BHD3 region of XPC, we conclude that the observed transient interactions involving the TGD and BHD1 motifs serve to guide the β-hairpin subdomain into the substrate double helix. Such an insertion occurs at a substantial energetic cost as it requires local disruption of base stacking and hydrogen bonds [53]. While 6-4PPs reduce the thermodynamic threshold of this conformational change by lowering the melting temperature of damaged DNA and, hence, allow for direct recognition by XPC, CPDs cause minimal DNA-destabilizing effects [57],[58]. Thus, the dependence on DDB2 for a β-hairpin insertion explains the exquisite defect of XP-E cells in repairing this more abundant type of UV lesion.

### Spatiotemporal DNA Repair Organization by UV-DDB

**Figure 7:**
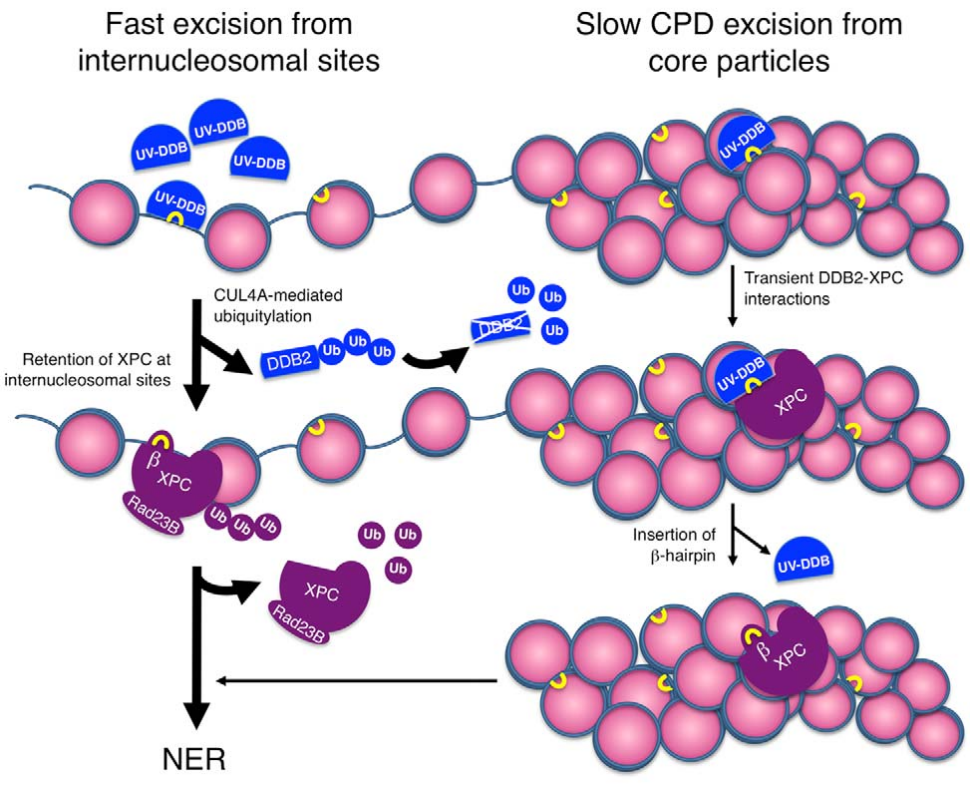
Novel regulatory principle in DNA repair. *Left*, ubiquitin-dependent prioritization of DNA repair to internucleosomal sites. The preferential UV-DDB accumulation on internucleosomal DNA leads to ubiquitylation of the XPC partner by CUL4A ligase. This modification promotes the XPC retention at internucleosomal sites, thus reducing its association with nucleosome core particles. The implementation of this ubiquitin code is required for the fast initial excision of UV lesions from internucleosomal DNA. Concomitantly ubiquitylated DDB2 is degraded, but XPC protein is protected from proteasome activity by RAD23B [47]. *Right*, ubiquitin-independent priming platform. UV-DDB undergoes very transient interactions with the TGD and BHD1 motifs of XPC, thereby facilitating the insertion of a β-hairpin subdomain, to hand over the substrate to the downstream NER process. This ubiquitin-independent substrate handover is required, regardless of nucleosome localization, for the excision of CPDs that on their own induce minimal distortions of the DNA duplex and, hence, are not recognizable by XPC alone. Ub, ubiquitin; β, β-hairpin of XPC. UV lesions are indicated with yellow brackets.

To summarize, UV-DDB exerts a bimodal action (Figure 7) to optimize the genome-wide NER reaction and ensure an initially fast (ubiquitin-dependent) removal of easily accessible lesions from internucleosomal DNA as well as the continued (ubiquitin-independent) excision of more intractable damage in nucleosome core particles. That an early (rapid) phase of repair takes place in internucleosomal DNA has already been shown by monitoring nucleotide incorporations into MNase-sensitive sites [59]. On the one hand, as illustrated in Figure 7, UV-DDB interrogates the chromatin to locate high-priority internucleosomal hotspots amenable to rapid excision. On the other hand, the DDB2 subunit of UV-DDB acts as a dynamic platform for the proper engagement of XPC with recalcitrant CPD lesions. Lower eukaryotes lack DDB2 [60], indicating that this subunit becomes critical in vertebrates, where larger and more compacted genomes necessitate a spatiotemporal coordinator of UV lesion recognition. The finding that CUL4A plays an accessory role by triggering a wave of fast DNA repair focused on only a fraction of chromatin, i.e. internucleosomal linkers, also reconciles the conflicting results as to the function of this ubiquitin ligase in stimulating [30]–[32] or inhibiting [33] UV responses. Because the same ligase also regulates the cellular level of DNA repair proteins and other transactions including the division cycle [33], it is conceivable that an interference with CUL4A activity may yield opposing effects depending on the organism, cellular context, or genetic background.

## Materials and Methods

Additional experimental procedures are given in Text S1.

### Reagents

The 15-mer sequence 5′-ACAGCGGTTGCAGGT-3′, carrying a CPD, was synthesized from phosphoramidite precursors. The same 15-mer with a 6-4PP was produced by irradiation and liquid-chromatographic purification [61]. Control oligonucleotides (5′-ACAGCGGTTGCAGGT-3′) were synthesized by Microsynth. The siRNA directed to CUL4A (target sequence 5′-TTCGAAGGACATCATGGTTCA-3′), DDB2 (target sequence 5′-AGGGATCAAGCAGTTATTTGA-3′), and XPC (target sequence 5′-TAGCAAATGGCTTCTATCGAA-3′) were purchased from Qiagen. The siCTRL consists of a pool of scrambled siRNA with at least four mismatches for all sequences in the human genome. MG132 was obtained from Sigma-Aldrich and added to the culture medium 5 h before each assay, at a concentration of 10 µM. PYR-41 (Santa Cruz) was used at a concentration of 50 µM and added to the medium 5 h before the assays. Restriction enzymes and MNase were from New England Biolabs.

### Plasmids and Cloning

The human *DDB2* sequence was obtained from plasmid DDB2-GFP-C1 (a gift from Dr. S. Linn, University of California, Berkeley, USA) by *BamH*I restriction and inserted into the expression vectors p3XFLAG-CMV-14 (Sigma-Aldrich) and pmRFP1-C3 (Dr. Elisa May, University of Konstanz, Germany). To construct the DDB2_79–427_-FLAG fusion, *Nde*I sites were generated by mutagenesis of codons 1 and 78. Subsequently, codons 1–78 were removed by *Nde*I digestion. For the cloning of XPC truncations and deletions, *Nde*I restriction sites were generated at the appropriate positions of vector XPC-pEGFP-N3. XPC-RFP was cloned by insertion of the XPC sequence into vector pmRFP1-C3 using *Kpn*I and *Sma*I sites. All plasmids were sequenced (Microsynth) to exclude accidental mutations.

### Culture Conditions

HeLa, HEK293T, U2OS, and Chinese hamster ovary (CHO) cells V79 were grown in humidified incubators (37 °C, 5% CO_2_) using Dulbecco's modified Eagle's medium (DMEM) supplemented with 10% (v/v) fetal bovine serum (FBS; Gibco), 100 U/ml penicillin, and 0.1 mg/ml streptomycin. For XP-E fibroblasts, the FBS concentration was 15% (v/v). Mouse embryonic fibroblasts (MEFs) ts-20 were grown at 32 °C in DMEM with 10% (v/v) FBS. Stably corrected H38-5 cells were cultured at 37 °C with hygromycin (50 µg/ml) to maintain expression of the complementing E1 enzyme. These MEFs were transferred to the restrictive temperature (39 °C) 18 h prior to the experiments.

### UV Irradiation and Photoproduct Excision

After removal of medium, cells were rinsed with phosphate-buffered saline (PBS) and irradiated with the indicated doses of UV-C from a germicidal lamp (254 nm wavelength). The progressive excision of 6-4PPs and CPDs was monitored using commercial antibodies as described in Text S1. For local damage induction, a 5-µm polycarbonate filter (Millipore) presoaked in PBS was placed over the cells followed by irradiation with 100 J/m^2^. After removal of the filter, the cells were incubated in fresh medium before processing for chromatin dissection, immunocytochemistry, or FRAP-LD analyses.

### Nucleosome Dissection

A combined salt extraction and MNase treatment (Figure S1A) was applied to analyze the partitioning of NER proteins. On 10-cm culture dishes, 5×10^6^ cells were grown to confluence and UV-irradiated for up to 10 s. After the indicated post-irradiation times (between 1 min and 24 h), the dishes were transferred onto ice, the cells were washed twice with 10 ml ice-cold PBS and scraped into a 1.5-ml tube with 0.3 ml of NP-40 lysis buffer [25 mM Tris-HCl (pH 8.0), 0.3 M NaCl, 1 mM EDTA, 10% (v/v) glycerol, 1% (v/v) NP-40, 0.25 mM phenylmethylsulfonyl fluoride, and EDTA-free protease inhibitor cocktail (Roche)] [25]. After a 30-min incubation on a turning wheel, free proteins not bound to chromatin (supernatant 1 in Figure S1A) were recovered by centrifugation (15,000 g, 4 °C, 10 min) and the volume was adjusted to 500 µl using NP-40 lysis buffer. The remaining insoluble chromatin was washed twice with 0.5 ml ice-cold CS buffer [31] consisting of 20 mM Tris-HCl, pH 7.5, 100 mM KCl, 2 mM MgCl_2_, 1 mM CaCl_2_, 0.3 M sucrose, and 0.1% (v/v) Triton X-100. Next, the chromatin was resuspended in 40 µl CS buffer and, after the addition of 5 µl 10× reaction buffer [500 mM Tris-HCl (pH 7.9), 50 mM CaCl_2_], 1 µl of bovine serum albumin (BSA; 1 mg/ml) and MNase (4 U/µl in a volume of 50 µl), incubated at 37 °C for 20 min. MNase digestions were stopped by the addition of EDTA (5 mM) and the solubilized proteins (supernatant 2 in Figure S1A) were separated from insoluble core particles by centrifugation at 15,000 g (10 min, 4 °C). This core particle fraction was dissolved in 80 µl denaturing buffer [20 mM Tris-HCl, pH 7.4, 50 mM NaCl, 1 mM EDTA, 0.5% (v/v) NP-40, 0.5% (v/v) deoxycholate, and 0.5% (w/v) sodium dodecyl sulfate (SDS)] [62] and sonicated (1×12 s). Alternatively, to generate the supernatant 3 of Figure S1F, the insoluble core particles were dissolved without sonication in 50 mM Tris-HCl, pH 8.0, 0.05% (v/v) NP-40 and 2.5 M NaCl as reported [63]. To obtain MNase dose dependences, chromatin pellets were digested with increasing enzyme concentrations. For the subsequent electrophoretic analysis, DNA fragments were extracted using the QIAamp Blood Kit (QIAGEN), resolved on 2% agarose gels, and stained with ethidium bromide.

### Protein Pull-down Assay

Polypeptides of 135–204 residues fused to GST (GST-XPC_607–741_, GST-XPC_607–766_, GST-XPC_679–832_, and GST-XPC_428–633_) were cloned and expressed in *E. coli* as described [64]. These polypeptides (120 pmol) were incubated (1 h, 4 °C) with 25 µl glutathione-Sepharose beads in 500 µl washing buffer [50 mM Tris-HCl (pH 8.0), 1 mM EDTA, 1 mM dithriothreitol, 10% (v/v) glycerol, 0.5% (v/v) Nonidet P-40, 150 mM NaCl, and 200 g/ml bovine serum albumin] containing 0.5% (w/v) nonfat dry milk. In the experiments with DNA, UV-DDB (120 pmol) was pre-incubated (1 h, 4 °C) with the indicated amounts of undamaged or damaged duplexes in a separate tube containing 500 µl washing buffer. The bead suspension containing GST-tagged polypeptides were washed three times with 1 ml washing buffer and incubated with UV-DDB for 20 min at room temperature in a total volume of 500 µl. The beads were then washed 3 times with 1 ml washing buffer containing nonfat dry milk, twice with washing buffer without nonfat dry milk, resuspended in loading buffer, and resolved on 10% denaturing polyacrylamide gels.

### Live-cell Analysis of Protein Dynamics

FRAP-LD measurements were performed on a Leica TCS SP5 confocal microscope equipped with an Ar^+^ laser (488 nm) and 63× oil immersion lens. The assays were performed in a controlled environment at 37°C and a CO_2_ supply of 5%. Cells transfected with GFP or RFP constructs were UV-irradiated (254 nm, 100 J/m^2^) through 5-µm polycarbonate filters. After 15-min incubations in complete medium, regions of interest (ROIs) corresponding to sites of GFP accumulation were photobleached at 50% laser intensity to reduce their fluorescence to that of the surrounding nuclear background. Fluorescence recovery was monitored 10 times using 0.7 s intervals followed by 10 frames at 5 s and 6 frames at 20 s. The results were adjusted for overall bleaching by correction with a reference ROI of the same size monitored at each time point. The values were used to calculate ratios between the damaged area in the foci and the corresponding intensity before bleaching. In the data display, the first fluorescence measurement after photobleaching is set to 0, while all following data points are plotted as a function of time.

## Supporting Information

Figure S1 Localization of UV-DDB to 6-4PPs in MNase-hypersensitive internucleosomal DNA. (A) Flow diagram illustrating the chromatin analysis after removal of unbound proteins (supernatant 1) by salt extraction (0.3 M NaCl). The complete MNase digestion of internucleosomal linker DNA [1] releases solubilized internucleosomal proteins (supernatant 2) and a remaining insoluble fraction containing the majority of nucleosome core particles. DNA quantifications (see panel D) show that only a marginal quantity of core particles appear in the soluble supernatant. (B) Ethidium bromide staining of agarose gels demonstrating the gradual DNA digestion with increasing MNase concentrations. Saturation is reached at 4 U/µl, whereby the whole chromatin is converted to nucleosome core fragments of 147 bp. Lanes 1–7 and 9, analysis of DNA from whole (“W”) chromatin; lane 8, 100-bp size markers; lane 10, insoluble fraction (“I”) containing most core fragments. (C) Quantification of UV lesions by enzyme-linked immunosorbent assay (ELISA). Exactly the same amounts (200 ng for 6-4PP detection and 10 ng for CPD detection, in duplicates) of whole genomic DNA (“Total DNA”), MNase-solubilized core fragments (“Sol. frag.”), or MNase-insoluble core fragments (“I. cores”) were analyzed in a microtiter plate using antibodies against 6-4PPs or CPDs. Control wells contained undamaged DNA. (D) Relative distribution of DNA, 6-4PPs, and CPDs in the different chromatin fractions resulting from MNase digestion (4 U/µl). The proportion of DNA and photolesions in digested internucleosomal DNA was calculated by subtraction from the respective values obtained with whole genomic DNA. (E) Preferential binding of UV-DDB to internucleosomal sites of UV-irradiated HeLa cells (30 J/m^2^) evidenced by an MNase dose response. The DDB2 binding to either insoluble core particles (“I. cores”) or solubilizable internucleosomal sites (“S. inter.”) was monitored by Western blotting, with the numbers in parentheses indicating the proportion of each fraction loaded onto the gel. (F) Solubilization of XPC associated with core particles by high-salt treatment. The MNase-resistant core particle fraction was incubated with buffer containing 2.5 M NaCl. By centrifugation, supernatant 3 was separated from the residual pellet and analyzed with antibodies against XPC and histone H3.
(TIF)

Figure S2 Ubiquitin-dependent DDB2 degradation and XPC positioning. (A) Immunoblot of HeLa whole-cell lysates visualizing the UV-dependent breakdown of DDB2 [2],[3]. This degradation is blocked by the proteasome inhibitor MG132 (10 µM). (B) Immunoblots of HeLa whole-cell lysates illustrating the degree of DDB2, XPC, or CUL4A depletion by transfection with the indicated siRNA reagents. CTRL, control siRNA. (C) Defective XPC ubiquitylation following DDB2 or CUL4A depletion by transfection of HeLa cells with specific siRNA (longer exposure of the internucleosomal fractions in Figure 3A and 3C, respectively). The asterisks denote the position of ubiquitylated XPC. (D) Abnormal nucleosome distribution of XPC in XP-E fibroblasts 1 h after UV exposure. The MNase digestion (4 U/µl) shows that essentially all of XPC is bound to the insoluble core particle fraction (“I. cores”). (E) Complementation of DDB2-depleted HeLa cells by transfection with a construct coding for DDB2-GFP. This transfection reconstitutes DDB2 expression and, hence, restores in part the ubiquitylation of XPC and its UV-dependent accumulation at internucleosomal sites (compare lanes 4 and 6).
(TIF)

Figure S3 Analysis of XPC positioning using small-molecule inhibitors. (A) Defective XPC ubiquitylation following treatment with the E1 inhibitor PYR-41 (50 µM, [4]): longer exposure of the internucleosomal fraction in Figure 3D. (B) Defective XPC ubiquitylation in HeLa cells treated with MG132 (10 µM; longer exposure of the internucleosomal fraction in Figure 3E). (C) Time course of DDB2 and XPC relocation to solubilizable internucleosomal sites of HeLa cells and differential effect of MG132. (D) DDB2 and XPC relocation to chromatin and effect of MG132 in p53-proficient U2OS cells. The MG132 concentration was 10 µM. See legend of Figure 1 for details.
(TIF)

Figure S4 Mapping of UV-DDB-XPC Interactions. (A) Time course of XPC-GFP relocation to UV-irradiated areas of DDB2-deficient CHO cells. GFP signals at UV lesion spots (*N* = 30) were quantified, normalized against the nuclear background, and expressed as a percentage of the 15-min time point; “+DDB2,” CHO cells complemented by transfection with a DDB2-RFP construct. (B) Domains of human XPC protein and truncation/deletion constructs used for the in situ mapping of DDB2-XPC interactions in chromatin. TGD, transglutaminase homology domain; BHD, β-hairpin domain. (C) Coimmunoprecipitation studies indicating that full-length DDB2 (DDB2_1–427_-FLAG) interacts with XPC-GFP in HEK293T cells. The lysates of doubly transfected cells were probed by the addition of anti-FLAG affinity beads and the resulting immunoprecipitates were analyzed by Western blotting using anti-FLAG and anti-GFP antibodies. (D) Truncated DDB2_79–427_-FLAG is unable to associate with DDB1 but still interacts with XPC-GFP. (E) DDB2 interacts with a polypeptide (XPC_520–633_-GFP) covering the TGD region of XPC protein. (F) DDB2 forms complexes with a polypeptide (XPC_607–831_-GFP) that displays the BHD region of XPC protein.
(TIF)

Figure S5 Measurement of DDB2 protein dynamics at UV lesions in chromatin. (A) Local fluorescence recovery rates [5],[6] demonstrating that the dissociation of DDB2 from lesion sites is accelerated by co-expression of human XPC. Foci of local DNA damage were generated by UV irradiation of CHO cells through micropore filters. The subsequent FRAP-LD analyses were performed in cells transfected with constructs coding for DDB2-GFP, either in the absence or in the presence of XPC-RFP (*N* = 15; error bars, s.e.m.). (B) The dissociation of DDB2-GFP from lesion sites is not affected by expression of the ΔHairpin-RFP construct that lacks amino acids 789–815 of the human XPC sequence (*N* = 15; error bars, s.e.m.).
(TIF)

Text S1 Supplementary material and methods.
(DOC)

## Abbreviations

6-4PP: 6-4 pyrimidine-pyrimidone photoproduct
BHD: β-hairpin domain
CPD: cyclobutane pyrimidine dimer
FRAP-LD: fluorescence recovery after photobleaching on local damage
GFP: green-fluorescent protein
NER: nucleotide excision repair
RFP: red-fluorescent protein
TGD: transglutaminase homology domain
UV: ultraviolet
UV-DDB: UV-damaged DNA-binding
XP: xeroderma pigmentosum
