# Supplementary figures and images for: Regulation of Nucleotide Excision Repair by UV-DDB: Prioritization of Damage Recognition to Internucleosomal DNA
Source: PLoS Biol. 2011 Oct 25;9(10):e1001183. doi: 10.1371/journal.pbio.1001183 (PMC3201922; doi:10.1371/journal.pbio.1001183)

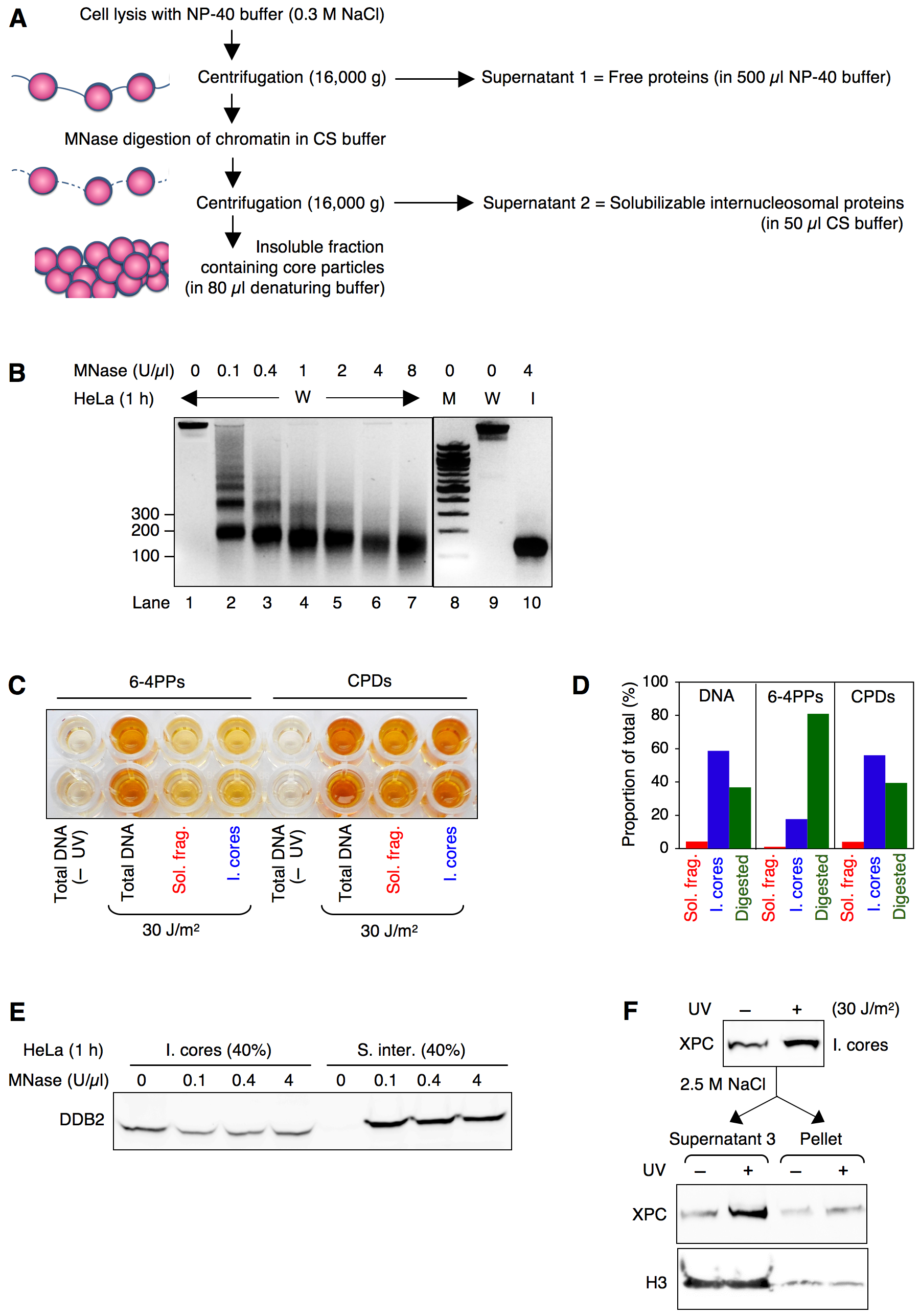

Supplement: Figure S1 — Localization of UV-DDB to 6-4PPs in MNase-hypersensitive internucleosomal DNA. (A) Flow diagram illustrating the chromatin analysis after removal of unbound proteins (supernatant 1) by salt extraction (0.3 M NaCl). The complete MNase digestion of internucleosomal linker DNA [1] releases solubilized internucleosomal proteins (supernatant 2) and a remaining insoluble fraction containing the majority of nucleosome core particles. DNA quantifications (see panel D) show that only a marginal quantity of core particles appear in the soluble supernatant. (B) Ethidium bromide staining of agarose gels demonstrating the gradual DNA digestion with increasing MNase concentrations. Saturation is reached at 4 U/µl, whereby the whole chromatin is converted to nucleosome core fragments of 147 bp. Lanes 1–7 and 9, analysis of DNA from whole (“W”) chromatin; lane 8, 100-bp size markers; lane 10, insoluble fraction (“I”) containing most core fragments. (C) Quantification of UV lesions by enzyme-linked immunosorbent assay (ELISA). Exactly the same amounts (200 ng for 6-4PP detection and 10 ng for CPD detection, in duplicates) of whole genomic DNA (“Total DNA”), MNase-solubilized core fragments (“Sol. frag.”), or MNase-insoluble core fragments (“I. cores”) were analyzed in a microtiter plate using antibodies against 6-4PPs or CPDs. Control wells contained undamaged DNA. (D) Relative distribution of DNA, 6-4PPs, and CPDs in the different chromatin fractions resulting from MNase digestion (4 U/µl). The proportion of DNA and photolesions in digested internucleosomal DNA was calculated by subtraction from the respective values obtained with whole genomic DNA. (E) Preferential binding of UV-DDB to internucleosomal sites of UV-irradiated HeLa cells (30 J/m2) evidenced by an MNase dose response. The DDB2 binding to either insoluble core particles (“I. cores”) or solubilizable internucleosomal sites (“S. inter.”) was monitored by Western blotting, with the numbers in parentheses indicating the p [file pbio.1001183.s001.tif]

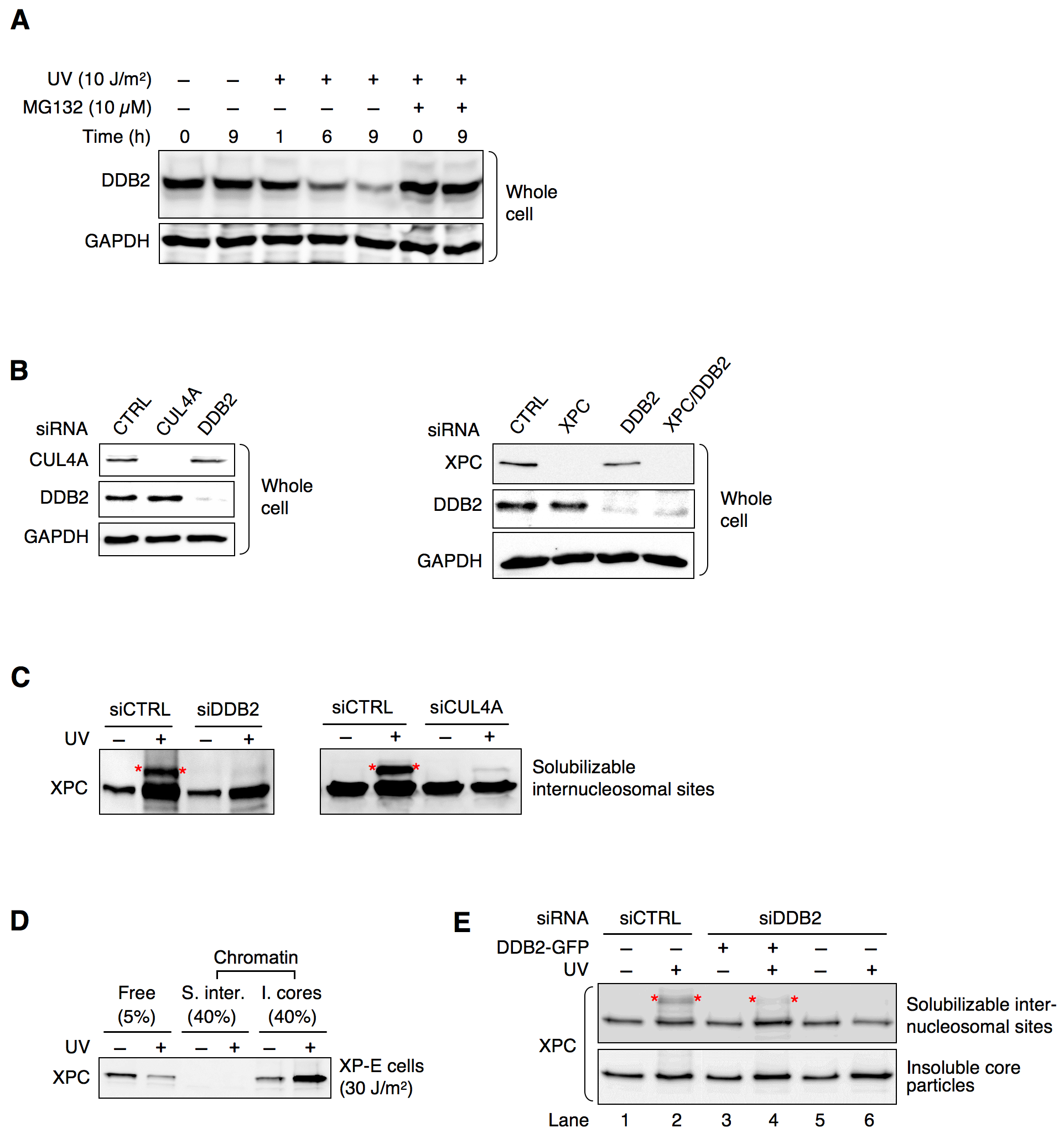

Supplement: Figure S2 — Ubiquitin-dependent DDB2 degradation and XPC positioning. (A) Immunoblot of HeLa whole-cell lysates visualizing the UV-dependent breakdown of DDB2 [2],[3]. This degradation is blocked by the proteasome inhibitor MG132 (10 µM). (B) Immunoblots of HeLa whole-cell lysates illustrating the degree of DDB2, XPC, or CUL4A depletion by transfection with the indicated siRNA reagents. CTRL, control siRNA. (C) Defective XPC ubiquitylation following DDB2 or CUL4A depletion by transfection of HeLa cells with specific siRNA (longer exposure of the internucleosomal fractions in Figure 3A and 3C, respectively). The asterisks denote the position of ubiquitylated XPC. (D) Abnormal nucleosome distribution of XPC in XP-E fibroblasts 1 h after UV exposure. The MNase digestion (4 U/µl) shows that essentially all of XPC is bound to the insoluble core particle fraction (“I. cores”). (E) Complementation of DDB2-depleted HeLa cells by transfection with a construct coding for DDB2-GFP. This transfection reconstitutes DDB2 expression and, hence, restores in part the ubiquitylation of XPC and its UV-dependent accumulation at internucleosomal sites (compare lanes 4 and 6). (TIF) [file pbio.1001183.s002.tif]

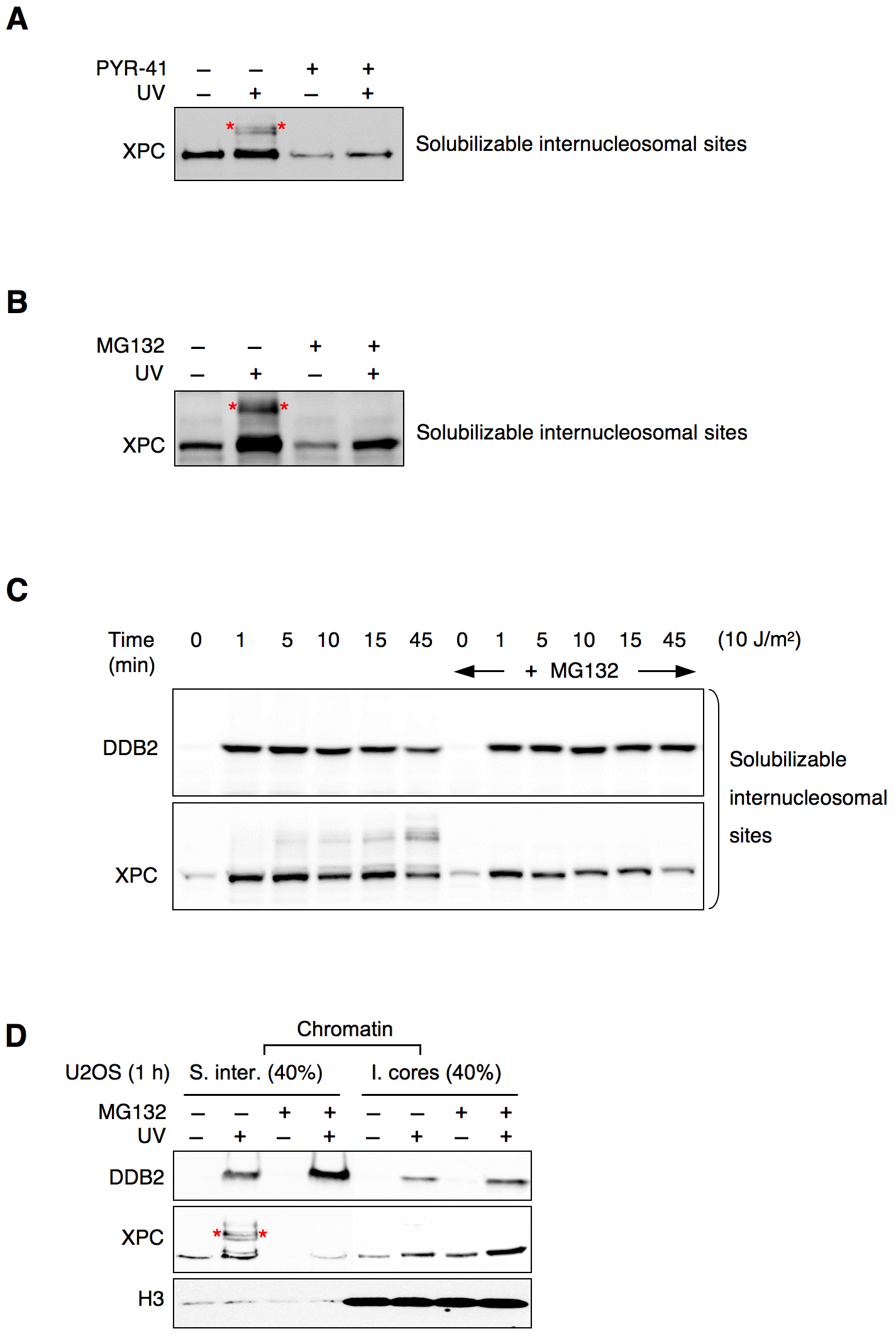

Supplement: Figure S3 — Analysis of XPC positioning using small-molecule inhibitors. (A) Defective XPC ubiquitylation following treatment with the E1 inhibitor PYR-41 (50 µM, [4]): longer exposure of the internucleosomal fraction in Figure 3D. (B) Defective XPC ubiquitylation in HeLa cells treated with MG132 (10 µM; longer exposure of the internucleosomal fraction in Figure 3E). (C) Time course of DDB2 and XPC relocation to solubilizable internucleosomal sites of HeLa cells and differential effect of MG132. (D) DDB2 and XPC relocation to chromatin and effect of MG132 in p53-proficient U2OS cells. The MG132 concentration was 10 µM. See legend of Figure 1 for details. (TIF) [file pbio.1001183.s003.tif]

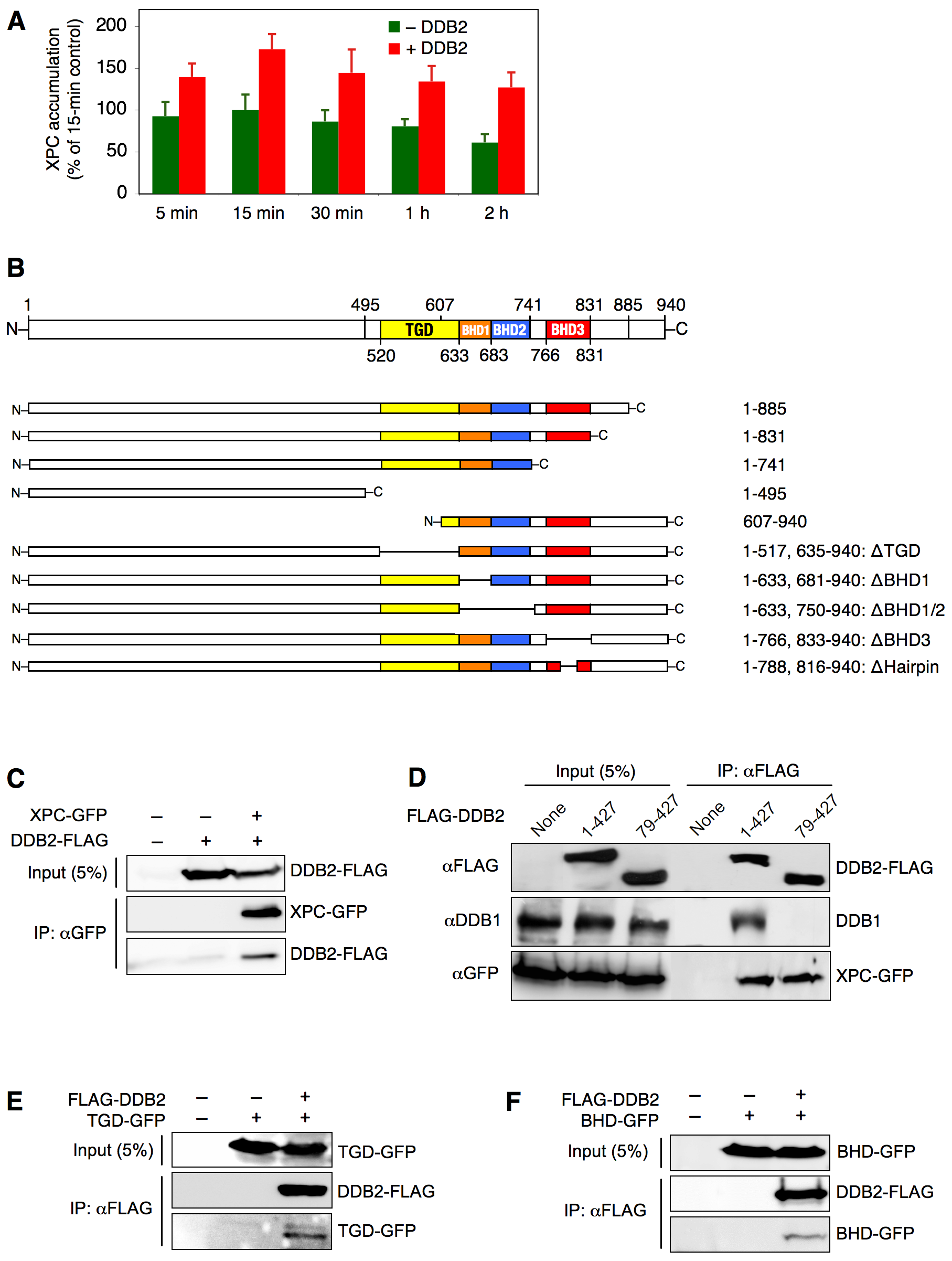

Supplement: Figure S4 — Mapping of UV-DDB-XPC Interactions. (A) Time course of XPC-GFP relocation to UV-irradiated areas of DDB2-deficient CHO cells. GFP signals at UV lesion spots (N = 30) were quantified, normalized against the nuclear background, and expressed as a percentage of the 15-min time point; “+DDB2,” CHO cells complemented by transfection with a DDB2-RFP construct. (B) Domains of human XPC protein and truncation/deletion constructs used for the in situ mapping of DDB2-XPC interactions in chromatin. TGD, transglutaminase homology domain; BHD, β-hairpin domain. (C) Coimmunoprecipitation studies indicating that full-length DDB2 (DDB21–427-FLAG) interacts with XPC-GFP in HEK293T cells. The lysates of doubly transfected cells were probed by the addition of anti-FLAG affinity beads and the resulting immunoprecipitates were analyzed by Western blotting using anti-FLAG and anti-GFP antibodies. (D) Truncated DDB279–427-FLAG is unable to associate with DDB1 but still interacts with XPC-GFP. (E) DDB2 interacts with a polypeptide (XPC520–633-GFP) covering the TGD region of XPC protein. (F) DDB2 forms complexes with a polypeptide (XPC607–831-GFP) that displays the BHD region of XPC protein. (TIF) [file pbio.1001183.s004.tif]

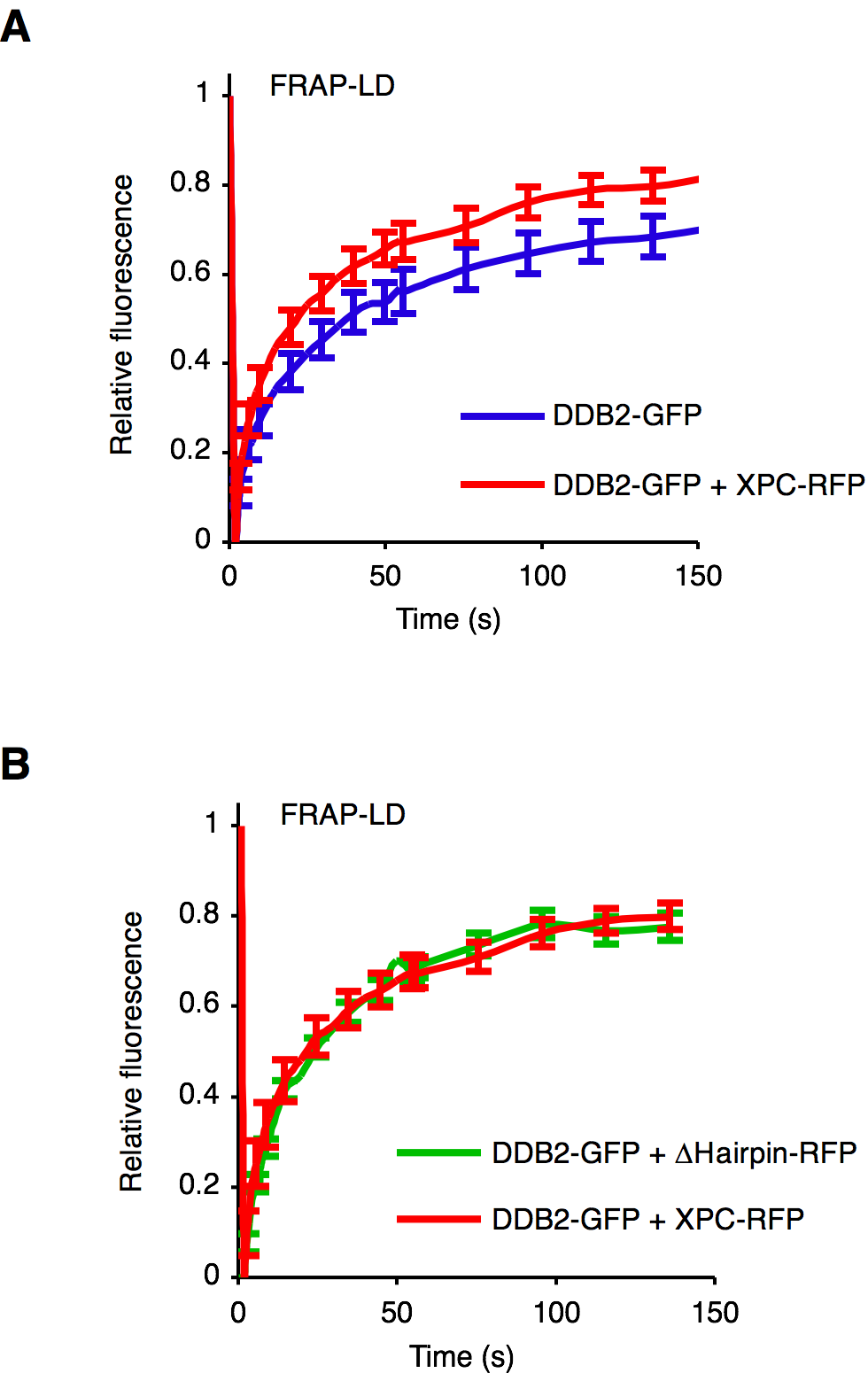

Supplement: Figure S5 — Measurement of DDB2 protein dynamics at UV lesions in chromatin. (A) Local fluorescence recovery rates [5],[6] demonstrating that the dissociation of DDB2 from lesion sites is accelerated by co-expression of human XPC. Foci of local DNA damage were generated by UV irradiation of CHO cells through micropore filters. The subsequent FRAP-LD analyses were performed in cells transfected with constructs coding for DDB2-GFP, either in the absence or in the presence of XPC-RFP (N = 15; error bars, s.e.m.). (B) The dissociation of DDB2-GFP from lesion sites is not affected by expression of the ΔHairpin-RFP construct that lacks amino acids 789–815 of the human XPC sequence (N = 15; error bars, s.e.m.). (TIF) [file pbio.1001183.s005.tif]
