# Supplementary material for: Regulation of Nucleotide Excision Repair by UV-DDB: Prioritization of Damage Recognition to Internucleosomal DNA
Source: PLoS Biol. 2011 Oct 25;9(10):e1001183. doi: 10.1371/journal.pbio.1001183 (PMC3201922; doi:10.1371/journal.pbio.1001183)
Supplement: Text S1 — Supplementary material and methods. (DOC) [file pbio.1001183.s006.doc]

**Supporting Information for**

**Regulation of Nucleotide Excision Repair by UV-DDB: Prioritization of Damage Recognition to Internucleosomal DNA**

Jia Fei, Nina Kaczmarek, Andreas Luch, Andreas Glas, Thomas Carell, Hanspeter Naegeli

**Text S1**

**Supplementary Materials and Methods**

**Proteins and antibodies**

The recombinant human DDB1-DDB2 heterodimer (containing His_6_-tagged DDB2) was kindly provided by Dr. A. Scrima and Dr. N. Thomä (FMI, Basel, Switzerland). Antibodies against the following proteins were used for Western blots following the manufacturers’ recommendations: human DDB2 (dilution 1:50, ab51017, Abcam), human XPC (1:1,000, ab6264, Abcam), mouse XPC (1:100, sc-74411, Santa Cruz), human RAD23A (1:500, ab3836, Abcam), human RAD23B (1:500, HPA029718, Sigma-Aldrich; 1:500, ab70602, Abcam), human XPB (1:200, sc-293, Santa Cruz), human XPG (1:500, X1629, Sigma-Aldrich), human XPA (1:100, sc-853, Santa Cruz), human GAPDH (1:4,000, No. 4300, Ambion), human histone H1.0 (1:1,000, ab11079, Abcam), human H3 (1:10,000, No. 07-690, Millipore) and H3K9m3 (1:10,000, No. 17-625, Millipore), human HP1 (1:200, sc-28735, Santa Cruz), human DDB1 (1:4,000, No. 612488, BD Bioscience), human CUL4A (1:1,000, ab34897, Abcam), FLAG M2 (1:4,000, F3165, Sigma-Aldrich) and GFP (1:4,000, No. 632375, Clontech). Horseradish peroxidase (HRP)-conjugated secondary antibodies against mouse or rabbit IgG were from Sigma-Aldrich, and HRP-conjugated antibodies against the His_6_ sequence (1:500, sc-8036) were from Santa Cruz.

**Sources of cell lines**

HeLa, HEK293T, U2OS and the Chinese hamster ovary (CHO) cells V79 were obtained from Dr. G. Marra, University of Zurich, Switzerland. Human XP-C (GM16093) and XP-E fibroblasts (GM02415) were purchased from the Coriell Institute for Medical Research (Camden, New Jersey, USA). The mouse embryonic fibroblast (MEF) cell line ts-20, thermosensitive for the ubiquitin-activating enzyme E1 , and stably corrected H38-5 cells were kindly provided by Dr. C. Borner, University of Freiburg, Germany and Dr. H. Ozer, New Jersey Medical School, USA.

**Transfections**

For gene silencing, 6 x 10^5^ HeLa cells were seeded into a 10-cm cell culture dish. Transfections with siRNA were carried out 72 h before the experiments, using Lipofectamine RNAiMAX reagent (Invitrogen) according to the manufacturer’s protocol. The siRNA final concentration was 15 nM. To complement silenced DDB2, HeLa cells were first transfected with siDDB2 and, 48 h later, transfected with the DDB2-GFP-C1 plasmid (5 µg) using the FuGENE HD reagent (Roche). Experiments were conducted following another 24-h incubation period, at a cell confluence of 90-95%. For immunoprecipitations, 6 x 10^6^ HEK293T cells were transfected in 10-cm dishes with 2.5 µg FLAG-DDB2 and 2.5 µg XPC-GFP vectors using the FuGENE reagent. For immunocytochemistry and live-cell imaging experiments, 6 x 10^5^ CHO cells were seeded into 6-well plates containing glass cover slips and transfected (FuGENE) with 1 µg each of the XPC-GFP, XPC-RFP, DDB2-GFP or DDB2-RFP vectors .

**Quantification of UV lesions**

Antibodies against 6-4PPs and CPDs (MBL International Corporation) were used in an enzyme-linked immunosorbent assay (ELISA) to quantify UV lesions according to the manufacturer’s instructions. Briefly, DNA purified from each nucleosome fraction (obtained by MNase digestion) and from the whole genome (before MNase digestion) was denatured by heating (95ºC, 10 min) followed by a 15-min incubation in ice-cold water. A volume of 50 µl per well of denatured DNA (4 µg/ml for 6-4PP detection, 200 ng/ml for CPD detection) was distributed into a 96-well microtiter plate coated with protamine sulfate (BD Biosciences) and dried overnight at 37ºC. The DNA-coated plates were washed five times with PBST [0.05% (v/v) Tween-20 in PBS] and blocked with 2% FBS in PBS at 37ºC for 30 min. The antibodies against either 6-4PPs (64M-2) or CPDs (TDM-2) were used for 30 min (37ºC) at dilution of 1:2,000 and 1:5,000, respectively. Primary antibodies bound to DNA molecules were recognized by biotin-labeled F(ab´)_2_ fragments of anti-mouse IgG (dilution 1:2,000; Invitrogen) added for 30 min at 37ºC. After washing the plates, 100 µl of a peroxidase-streptavidin conjugate (dilution 1:10,000; Invitrogen) was distributed into each well. The reaction was started by adding 0.5 mg/ml o-phenylenediamine, 0.007% H_2_O_2_ and 0.1 M citrate-phosphate buffer (pH 5.0), stopped with 50 µl of 2 M H_2_SO_4_, and monitored by measuring the absorbance at 490 nm in a PLUS384 microplate spectrophotometer (Molecular Devices).

**Immunoprecipitation**

One day after transfection, the medium was aspirated and HEK293T cells were washed twice with ice-cold PBS and harvested by scraping in 0.5 ml of ice-cold NP-40 lysis buffer. After 30 min, the cells were snap frozen in liquid nitrogen and the resulting lysates were centrifuged at 10,000 g for 10 min at 4°C. The supernatant was mixed with 30 μl anti-FLAG M2 affinity gel (Sigma) and incubated under rotation overnight at 4°C. After two washes each with ice-cold TNT buffer [50 mM Tris-HCl (pH 7.5), 140 mM NaCl, 1% (v/v) Triton X-100] and TN buffer [50 mM Tris-HCl (pH 7.5), 140 mM NaCl], the immunoprecipitates were eluted with 0.5 mg/ml 3xFLAG peptides (Sigma), boiled in loading buffer [60 mM Tris-HCl, pH 6.8, 2% (w/v) SDS, 10% (v/v) glycerol, 4% (v/v) 2-mercaptoethanol and 0.002% (w/v) bromophenol blue] and resolved by denaturing polyacrylamide gel electrophoresis.

**Immunoblotting**

The electrophoretically resolved samples were transferred to a polyvinylidene (PVDF) membrane (BioRad) that was blocked by incubation for 2 h at room temperature with TBST buffer [Tris-buffered saline with 0.05% (v/v) Tween-20] containing 5% (w/v) nonfat dry milk. Primary antibodies were used at the indicated dilutions in TBST containing 2.5% (w/v) nonfat dry milk. HRP-conjugated secondary antibodies were diluted 10,000-fold in TBST containing 2.5% nonfat dry milk. Reactions were developed with SuperSignal West Pico or Femto substrate (Pierce) and documented with a FUJI LAS-3000 imaging system. The resulting data were quantified using the Quantity One software (BioRad) to calculate mean values and standard deviations from the blots of at least three independent experiments. All data were finally corrected for the different loading volumes during the electrophoretic separation.

**Calculations used for quantitative assessments of nucleosome partitioning**

The nucleosome partitioning of DDB2 and XPC was determined from non-saturated immunoblot signals quantified using the Quantity One Software (BioRad). To correct for the distinct volumes of each fraction and the respective loadings on denaturing polyacrylamide gels, the partitioning between free (F) proteins, not bound to chromatin, and the fractions of either solubilizable internucleosomal sites (S) or insoluble nucleosome core particles (I) was determined using the following equation: F x 20 + S x 2.5 + I x 2.5 = Total (100%). This calculation reflects the fact that only 5% of free proteins, but 40% of the solubilizable or insoluble MNase fractions were loaded onto the gels. For the experiments of Figures 3A, 3C, 3D and 3E, this equation was: F x 20 + S x 1.5 + I x 3 = Total (100%), thus reflecting that in these depletion or inhibition experiments a higher proportion of the solubilizable internucleosomal sites, relative to insoluble core particles, was loaded onto the gels.

**Immunocytochemistry**

Following a 15-min incubation after local UV damage induction, the medium was aspirated, the cells were rinsed with PBS and fixed for 15 min at room temperature using 4% (v/v) paraformaldehyde (Sigma-Aldrich) in PBS. The cells were then permeabilized twice with PBS containing 0.1% (v/v) Tween-20 for 10 min and DNA was denatured with 0.07 M NaOH for 8 min. Next, the samples were washed three times with 0.1% Tween-20 and incubated (30 min, 37°C) with 20% FBS in PBS to inhibit unspecific binding. The samples were incubated (1 h at 37°C in 5% FBS) with primary antibodies directed against CPDs (TDM-2; dilution 1:1’000). The samples were then washed with 0.1% Tween-20, blocked twice for 10 min with 20% FBS, and treated with Alexa Fluor 594 dye-conjugated secondary antibodies (Invitrogen; dilution 1:400) for 30 min at 37°C. After washing with 0.1% Tween-20 in PBS, the nuclei were stained for 10 min with Hoechst dye 33258 (200 ng/ml). Finally, the samples were washed three times and analyzed in a Leica SP5 confocal microscope equipped with a 63x oil immersion lens. For quantification of the immunocytochemistry results, a minimum of 30 cells were analyzed per experiment.

These immunocytochemical analyses were used to select a range of XPC-GFP expression that is comparable to the level of endogenous XPC in human cell lines . Only cells expressing such low levels of XPC-GFP in the normal physiologic range were used for FRAP-LD experiments.

### References

1. Telford DJ, Stewart BW (1989) Micrococcal nuclease: its specificity and use for chromatin analysis. Int J Biochem 21: 127-137.

2. Moser J, Volker M, Kool H, Alekseev S, Vrieling H, et al. (2005) The UV-damaged DNA binding protein mediates efficient targeting of the nucleotide excision repair complex to UV-induced photolesions. DNA Repair (Amst) 4: 571-582.

3. Rapic-Otrin V, McLenigan MP, Bisi DC, Gonzalez M, Levine AS (2002) Sequential binding of UV DNA damage binding factor and degradation of the p48 subunit as early events after UV irradiation. Nucleic Acids Res 30: 2588-2598.

4. Yang Y, Kitagaki J, Dai R-M, Tsai YC, Lorick KL, et al. (2007) Inhibitors of ubiquitin-activating enzyme (E1), a new class of potential cancer therapeutics. Cancer Res 67: 9472-9481.

5. Luijsterburg MS, Goedhart J, Moser J, Kool H, Geverts B, et al. (2007) Dynamic in vivo interaction of DDB2 E3 ubiquitin ligase with UV-damaged DNA is independent of damage-recognition protein XPC. J Cell Sci 120: 2706-2716.

6. Alekseev S, Luijsterburg MS, Pines A, Geverts B, Mari PO, et al. (2008) Cellular concentrations of DDB2 regulate dynamic binding of DDB1 at UV-induced DNA damage. Mol Cell Biol 28: 7402-7413.

7. Chowdary DR, Dermody JJ, Jha KK, Ozer HL (1994) Accumulation of p53 in a mutant cell line defective in the ubiquitin pathway. Mol Cell Biol 14: 1997-2003.

8. Camenisch U, Trautlein D, Clement FC, Fei J, Leitenstorfer A, et al. (2009) Two-stage dynamic DNA quality check by xeroderma pigmentosum group C protein. EMBO J 28: 2387-2399.
